# Nap1 and Kap114 co-chaperone H2A-H2B and facilitate targeted histone release in the nucleus

**DOI:** 10.1083/jcb.202408193

**Published:** 2024-11-27

**Authors:** Ho Yee Joyce Fung, Jenny Jiou, Ashley B. Niesman, Natalia E. Bernardes, Yuh Min Chook

**Affiliations:** 1 Department of Pharmacology, University of Texas Southwestern Medical Center, Dallas, TX, USA; 2 Department of Biophysics, University of Texas Southwestern Medical Center, Dallas, TX, USA

## Abstract

Core histones, synthesized and processed in the cytoplasm, must be chaperoned as they are transported into the nucleus for nucleosome assembly. The importin Kap114 transports H2A-H2B into the yeast nucleus, where Ran^GTP^ facilitates histone release. Kap114 and H2A-H2B also bind the histone chaperone Nap1, but how Nap1 and Kap114 cooperate in transport and nucleosome assembly remains unclear. Here, biochemical and structural analyses show that Kap114, H2A-H2B, and a Nap1 dimer (Nap1_2_) associate in the absence and presence of Ran^GTP^ to form equimolar complexes. A previous study had shown that Ran^GTP^ reduces Kap114’s ability to chaperone H2A-H2B, but a new cryo-EM structure of the Nap1_2_•H2A-H2B•Kap114•Ran^GTP^ complex explains how both Kap114 and Nap1_2_ interact with H2A-H2B, restoring its chaperoning within the assembly while effectively depositing it into nucleosomes. Together, our results suggest that Kap114 and Nap1_2_ provide a sheltered path that facilitates the transfer of H2A-H2B from Kap114 to Nap1_2_, ultimately directing its specific deposition into nucleosomes.

## Teaser

It was unclear how the H2A-H2B chaperone Nap1 cooperates with the importin Kap114 to transport and process H2A-H2B. Fung et al. explain how they co-chaperone H2A-H2B, and how Ran^GTP^ and Nap1, within the quaternary Nap1_2_/H2A-H2B/Kap114/Ran^GTP^ complex, coordinate the transfer of H2A-H2B from Kap114 to assembling nucleosomes.

## Introduction

Formation of new nucleosomes to pack newly replicated DNA during the S-phase of the cell cycle begins with the rapid synthesis of core histones H3, H4, H2A, and H2B, followed by their swift transport into the nucleus. Exposure of these very basic polypeptides to the cellular environment is deeply deleterious as they form toxic aggregates easily (Hogan and Foltz, 2021; Singh et al., 2010). Therefore, core histones are thought to be always chaperoned and never free (Elsässer and D’Arcy, 2013; Hammond et al., 2017). As H3 and H4 emerge from translating ribosomes, they are folded into H3-H4 heterodimers by heat shock proteins and folding chaperones, acetylated at several lysine side chains (Benson et al., 2006; Campos et al., 2010; Tagami et al., 2004), and then passed to histone chaperone ASF1 and *H. sapiens* (*Hs*) Importin-4 or its *S. cerevisiae* (*Sc*) homolog Kap123 for transport across the nuclear pore complex (NPC) into the nucleus (Baake et al., 2001; Bernardes and Chook, 2020; Mühlhäusser et al., 2001; Schwamborn et al., 1998).

Unfortunately, the steps of H2A-H2B biosynthesis and processing have not been delineated. However, selective ribosome profiling studies showed no association of *Sc* importins with the nascent chains of H2A and H2B (Seidel et al., 2023), suggesting that H2A-H2B processing may also involve interaction with heat shock proteins and histone chaperones prior to importin-binding for nuclear import. The heat shock proteins/folding chaperones for H2A and H2B have not been identified, and the only known H2A-H2B histone chaperone in the *Sc* cytoplasm is nucleosome assembly protein 1 or Nap1 (Chen et al., 2016; Huang et al., 2020; Mosammaparast et al., 2002). H2A-H2B also binds several *Hs* Nap1 homologs in human cells (Chang et al., 1997; Okuwaki et al., 2010). The importin primarily responsible for H2A-H2B nuclear import is also known. Multiple studies have reported on H2A and H2B import by the homologous and orthologous importins *Sc* Karyopherin-114 (Kap114) and *Hs* Importin-9 (IMP9/IPO9) (Baake et al., 2001; Jäkel et al., 2002; Jiou et al., 2023; Johnson-Saliba et al., 2000; Kimura et al., 2017; Mosammaparast et al., 2001, 2002; Mühlhäusser et al., 2001; O’Reilly et al., 2011; Padavannil et al., 2019).

**Figure 1.**
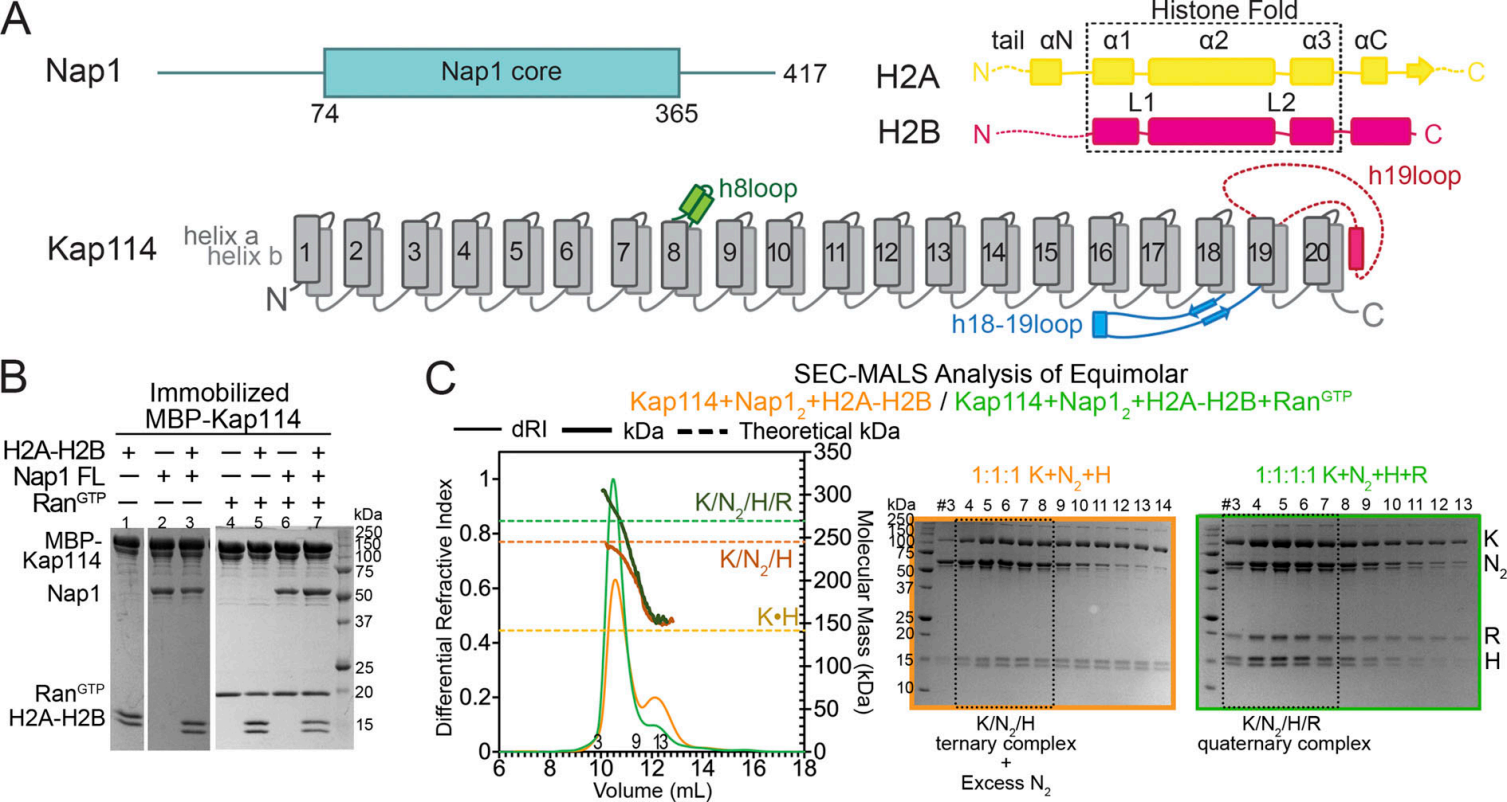
**Interactions between Kap114, Nap1, H2A-H2B, and Ran**^**GTP**^**. (A)** Organization schematics of the Nap1 (cyan), H2A (yellow), H2B (magenta), and Kap114 (gray, long loops labeled) polypeptides. **(B)** Pull-down assay with immobilized MBP-Kap114 (1 µM) ± equimolar Nap1_2_ ± Ran^GTP^. **(C)** SEC-MALS analysis of equimolar Kap114, Nap1_2_, and H2A-H2B mixtures without (orange) and with Ran^GTP^ (green). Left panel: differential refractive index (dRI, left y-axis, thin lines) and molecular mass (kDa, right y-axis, thick lines) traces, with theoretical masses of the Kap114•H2A-H2B (K•H), Kap114/Nap1_2_/H2A-H2B (K/N_2_/H), and Kap114/Nap1_2_/H2A-H2B/Ran^GTP^ (K/N_2_/H/R) complexes marked with dashed lines. Right panels: Coomassie-stained SDS-PAGE of peak fractions. When Ran^GTP^ is absent, the presence of a minor peak that matches K•H suggests the likely presence of some free Nap1 oligomers. Controls in Figs. S1 and S2. Source data are available for this figure: SourceData F1.

H2A and H2B contain disordered N- and C-terminal tails and central alpha helices that fold together into the globular H2A-H2B histone-fold domain, with very basic surfaces that bind DNA in the nucleosome (Fig. 1 A) (Luger et al., 1997). These same surfaces are shielded when H2A-H2B binds the *Sc* Nap1 dimer (Nap1_2_), which is mostly localized to the yeast cytoplasm where it chaperones newly synthesized and folded H2A-H2B (Aguilar-Gurrieri et al., 2016; Calvert et al., 2008; Mosammaparast et al., 2002; Park et al., 2008). Nap1 also has well-known roles in the nucleus, including nucleosome assembly/remodeling, transcription, DNA repair, and mitosis, suggesting that it may shuttle between the nucleus and the cytoplasm (Aguilar-Gurrieri et al., 2016; Altman and Kellogg, 1997; Andrews et al., 2010; Chen et al., 2016; Del Rosario and Pemberton, 2008; Dronamraju et al., 2017; Hsu et al., 2019; Kellogg and Murray, 1995; Krajewski, 2020; Levchenko and Jackson, 2004; Mazurkiewicz et al., 2006; Moshkin et al., 2013; Nagae et al., 2023; Park et al., 2005; Sharma and Nyborg, 2008; Vlijm et al., 2012). Genetic interaction of *Sc* Nap1 with Kap114 and with Ran binding proteins Yrb1 and Yrb2 have been reported, and the histone chaperone was also reported to be a cofactor for H2A-H2B nuclear import by Kap114 (Mosammaparast et al., 2002; Straube et al., 2010; Zlatanova et al., 2007). Furthermore, immunoprecipitation (IP) showed Kap114 association with both Nap1 and H2A-H2B in the yeast cytosolic extract and the Ran^GTP^-rich nuclear extract, suggesting that the three proteins are associated in both the cytoplasm and the nucleus (Mosammaparast et al., 2002, 2005).

The mode of H2A-H2B recognition by Kap114/IMP9 is well understood: the superhelical Kap114 wraps around the H2A-H2B histone-fold domain, occluding the histone’s DNA-binding surfaces and functioning as a histone chaperone (Jiou et al., 2023; Liao et al., 2020). Unlike most importin•cargo complexes, which are dissociated by the GTPase Ran^GTP^, Kap114•H2A-H2B forms a stable ternary complex with Ran^GTP^. This complex alters the interactions between Kap114 and H2A-H2B, facilitating histone release (Jiou et al., 2023). Although the interaction between Kap114 and H2A-H2B, both in the absence and presence of Ran^GTP^, is well characterized, the role of Nap1 as a co-import factor and its influence on nucleosome assembly remains unknown.

Here, we used biochemical analyses and cryo-EM structure determination to reveal the quaternary Nap1_2_•H2A-H2B•Kap114•Ran^GTP^ complex and its cytosolic counterpart. In this assembly, Ran^GTP^ binds to the N-terminal HEAT repeats of Kap114 while the H2A-H2B domain is sequestered by Nap1 and the C-terminal HEAT repeats of Kap114. DNA competition and nucleosome assembly assays confirmed that in the presence of Ran^GTP^, Kap114 and Nap1 cooperate to shield H2A-H2B from non-specific aggregation with DNA and transfer H2A-H2B effectively and specifically into nucleosomes.

## Results

### Interactions between Kap114, Nap1, H2A-H2B, and Ran^GTP^

**Figure S1.**
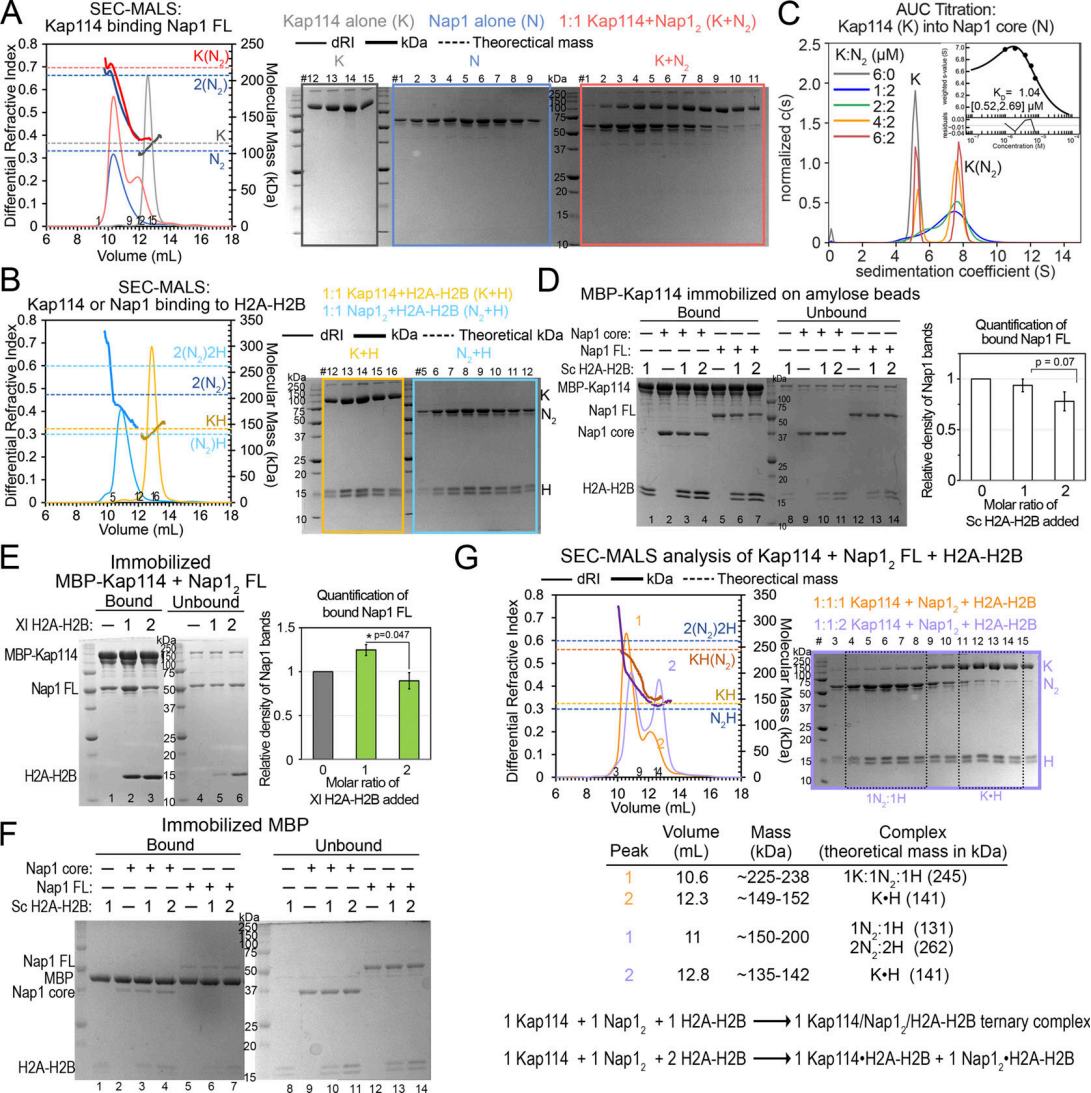
**Biochemical analysis of interactions between Kap114, Nap1, and H2A-H2B. (A)** SEC-MALS analysis of Nap1 FL (N; blue), Kap114 (K; gray), and a 1:1 mixture of both (red). Left panel: The differential refractive index (dRI) traces are plotted as thin lines (left y-axis) and the molecular mass (kDa) traces as thick lines (right y-axis). The theoretical masses of the indicated proteins are marked with dashed lines. Right panel, Coomassie-stained SDS-PAGE of peak fractions. Results: As previously reported, N alone formed tetramers with the apparent molecular mass of ∼200 kDa (elution volume ∼10.3 ml). K alone eluted at ∼12.5 ml with the expected apparent molecular mass ∼110 kDa. A 1:1 molar mixture of K and N_2_ formed a peak of ∼220 kDa that matches a K•N_2_ complex. The increase in DRI signal of the K+N_2_ compared to the N traces is consistent with incorporation of one K molecule. **(B)** SEC-MALS experiment for K (yellow) or N (cyan) binding to H2A-H2B (H) at the indicated molar ratios, plotted as in A. Results: K+H eluted with the expected molecular mass (elution volume ∼13 ml), whereas the N_2_+H mixture eluted at volumes that span molecular masses of 150–200 kDa, possibly due to a mixture of N_2_•H, N_2_•H_2_ and 2(N_2_•H) complexes. **(C)** AUC titration and binding isotherm (inset) of Kap114 (K; 5.2 S) into the Nap1 core dimer (N_2_) at the concentrations indicated. Molecular weight estimate, using the c(s) distribution of the most saturated 6:2 M ratio sample of the 7.9 S complex was 178 kDa, consistent with a K•N_2_ complex (theoretical molecular weight, 186 kDa). The isotherm was generated using a one-site binding model and fitting residuals are plotted below. The dissociation constant or K_D_ is shown with the values in brackets representing a 95% confidence interval. **(D)** The full gel of one of the two binding assays shown in Fig. 1 B: 1 µM immobilized MBP-Kap114 and Nap1_2_ core or FL ± 1 or 2 µM *Sc* H2A-H2B. Bound and unbound proteins after extensive washing were visualized by Coomassie-stained SDS-PAGE. Quantification of the average relative intensities of triplicate experiments of the bound FL Nap1, when normalized to the sample without H2A-H2B, is plotted with error bars that indicate standard deviation (SD). Unpaired, two-sided Student’s *t* test was performed. Data distribution was assumed to be normal but it was not formally tested. **(E)** Pull-down binding assay as in D, but with *Xl* H2A-H2B. Unlike *Sc* H2A-H2B, *X. laevis* (*Xl*) H2A-H2B increased *Sc* Nap1 association with Kap114, suggesting that different H2A-H2B homologs bind *Sc* Nap1 and Kap114 differently. Student t-test shows significant difference between 1 and 2 µM H2A-H2B samples, where less Nap1 was pulled down in the presence of excess H2A-H2B, suggesting destabilization of the ternary Kap114/Nap1_2_/H2A-H2B complex. **(F)** Control pull-down experiment of 1 µM MBP (immobilized) and equimolar Nap1_2_ ± H2A-H2B (1 or 2 M ratio). Background binding of Nap1 to the immobilized MBP was minimal. **(G)** SEC-MALS analysis of 1:1:1 (orange) or 1:1:2 (lilac) molar ratio K, N, and H mixtures, plotted as in A. Tabulated SEC-MALS results shown below. At 1:1:1 M ratio, most of the proteins assemble into a 1 Kap114/1 Nap1_2_/1 H2A-H2B complex. There is a minor population of Kap114•H2A-H2B in peak 2, and thus there must be some excess Nap1_2_ tetramers (∼200 kDa) in peak 1. When H2A-H2B is in excess; only Kap114•H2A-H2B, Nap1_2_/H2A-H2B complexes formed, as the peak centers match the two traces in B. In summary, both pull-down assays and SEC-MALS analysis support that excess H2A-H2B destabilizes a 1:1:1 Kap114/Nap1_2_/H2A-H2B ternary complex, dissociating it into binary Kap114•H2A-H2B and Nap12•H2A-H2B complexes. Ternary Kap114/Nap1_2_/H2A-H2B interactions, such as in the cytoplasm, maybe most stable when all H2A-H2B heterodimers are adequately chaperoned. Source data are available for this figure: SourceData FS1.

Previous reports of biochemical and structural studies of Kap114•H2A-H2B and Nap1_2_•H2A-H2B complexes, as well as pull-down studies of Kap114 and Nap1 from yeast lysates have informed on binary interactions between these proteins (Aguilar-Gurrieri et al., 2016; Jiou et al., 2023; Mosammaparast et al., 2002, 2005). Here, we confirm these findings using analytical ultracentrifugation (AUC) and size exclusion chromatography multiangle light scattering (SEC-MALS) analyses with purified recombinant Kap114, Nap1, and H2A-H2B proteins. Nap1 alone is a mixture of dimers and tetramers (Fig. S1 A), while Nap1 binds H2A-H2B to form a mixture of a Nap1 dimer (Nap1_2_) bound to one H2A-H2B (Nap1_2_•H2A-H2B) and larger Nap1/H2A-H2B assemblies (Fig. S1 B). Nap1 binds Kap114 to form a 1:1 Kap114:Nap1_2_ complex (Fig. S1, A and C).

Next, we focused on interactions between the Kap114, Nap1, and H2A-H2B, in the absence and presence of Ran^GTP^. Immobilized MBP-Kap114 pulled down Nap1 in the absence and presence of H2A-H2B; Kap114 also pulled down H2A-H2B in the absence and presence of Nap1 (Fig. 1 B and Fig. S1, D–F). SEC-MALS analysis showed the Kap114, Nap1_2_, and H2A-H2B together forming a complex that matches the theoretical mass of a 1:1:1 Kap114/Nap1_2_/H2A-H2B complex (Fig. 1 C and Fig. S1 G).

**Figure S2.**
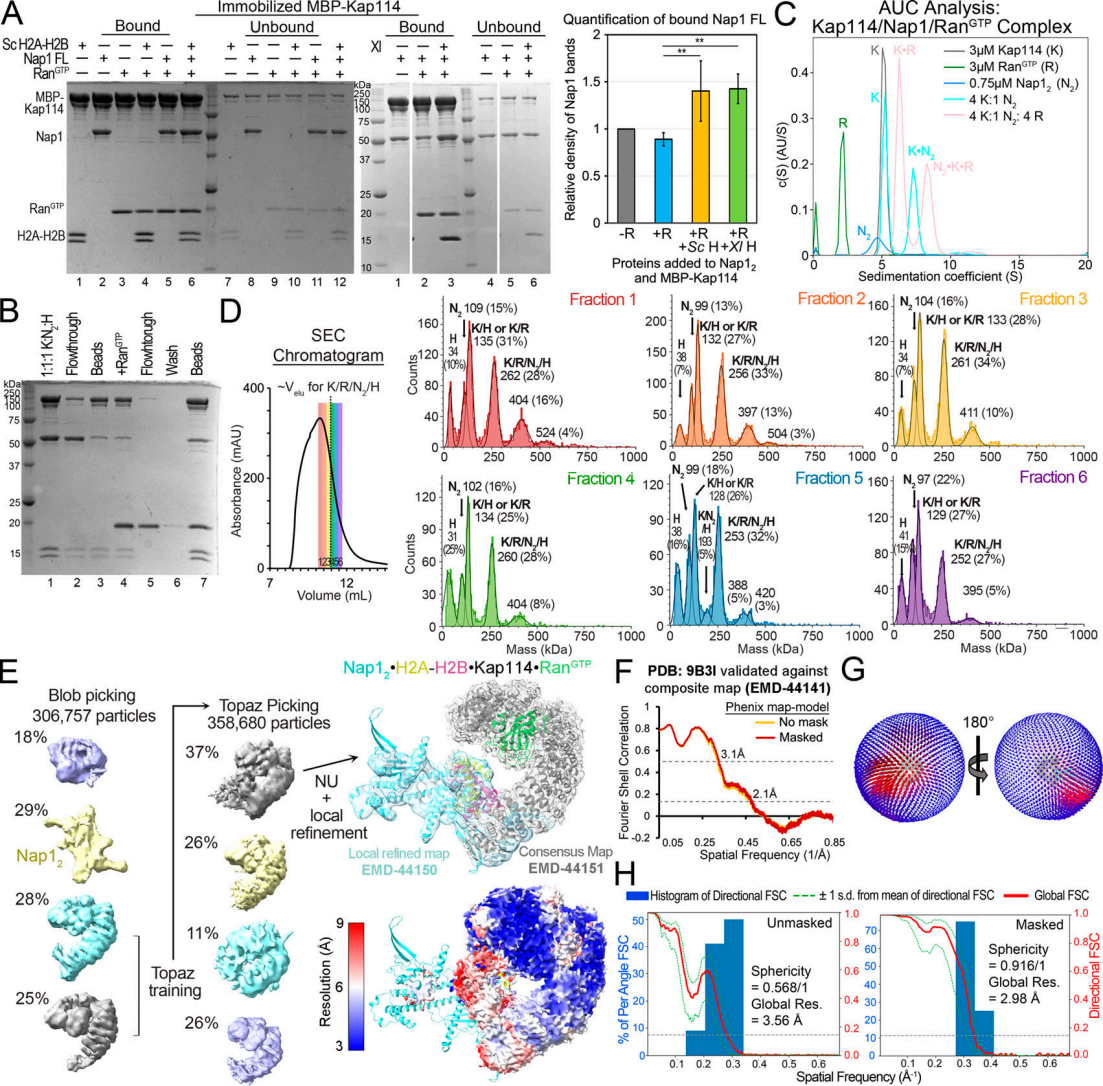
**Ran**^**GTP**^ **interaction with Kap114, Nap1**_**2**_**, and H2A-H2B mixtures. (A)** The full gel of one of the two binding assays shown in Fig. 1 B, which is also similar to assays in Fig. S1, D and E. MBP-Kap114 (1 µM) was immobilized with equimolar Nap1_2_ FL ± *Sc* or *Xl* H2A-H2B ± Ran^GTP^. Quantification of the Kap114-bound Nap1 band intensities from triplicate experiments is shown on the right. ** indicate P value <0.01. Student’s *t* test was performed two-tailed and unpaired. Data distribution was assumed to be normal but it was not formally tested. MBP-Kap114 pulled down more Nap1 when both H2A-H2B and Ran^GTP^ are present. This result is consistent with SEC-MALS data showing a smaller shoulder at the ∼12.2 ml peak of the Nap1_2_•H2A-H2B•Kap114•Ran^GTP^ trace (green; likely due to Kap114•H2A-H2B) than in the Nap1_2_•H2A-H2B•Kap114 trace (orange) in Fig. 1 C. **(B)** The same pull-down assay as in A, except 3 µM Ran^GTP^ was added after pre-assembly of the MBP-Kap114/Nap1_2_/H2A-H2B (K:N_2_:H) complex and immobilization on beads. Individual steps of the binding assay are visualized by SDS-PAGE. The quaternary Kap114/Nap1_2_/H2A-H2B/Ran^GTP^ complex formed regardless of the order of protein addition, and no Nap1 or H2A-H2B was dissociated by Ran^GTP^. **(C)** AUC analysis of Kap114-Nap1 interaction in the presence of Ran^GTP^. Plots of the c(s) distributions of unliganded proteins, Kap114 (K, 5.2 S), Nap1_2_ core (N_2_, 4.9 S) and Ran^GTP^ (R, 2.1 S), and mixtures of the proteins with indicated molar ratios. The species corresponding to the individual peaks are labeled. Ran^GTP^ binding increased sedimentation coefficient similarly for unliganded Kap114 (K, 5.2 → K•R, 6.3S) and for Kap114•Nap1_2_ (K•N_2_, 7.3 → N_2_•K•R, 8.3S). The Kap114•Ran^GTP^ and Nap1_2_•Kap114•Ran^GTP^ complexes had estimated molecular weights of 138 and 206 kDa, respectively, consistent with equimolar complexes. **(D)** Left: SEC chromatogram of the Kap114/Nap1_2_/H2A-H2B/Ran^GTP^ sample. Kap114, Nap1_2_, H2A-H2B, and Ran^GTP^ were mixed in equimolar ratio and dialyzed overnight before mild crosslinking. Fractions 1–6 of the SEC chromatogram are colored red to purple, and the typical elution volume of a non-crosslinked complex that contains all four proteins is marked with a dotted line. Right: Mass photometry traces of each of the six SEC fractions, with mean masses (kDa) and relative populations (%) indicated above the fitted gaussian peaks, along with the likely protein or complex that correspond to the approximate masses. Fraction 3, most enriched with the complex containing Kap114, Nap1_2_, H2A-H2B and Ran^GTP^, was used for cryo-EM grid preparation. The largest (∼380–410 kDa) species in each of the six SEC fractions may contain crosslinked complexes of K/R/N_2_/H with K•R; such a large assembly was not observed in AUC or SEC-MALS analyses where the proteins were not crosslinked. **(E)** Particle distribution of cryo-EM data obtained for the quaternary Kap114/Ran^GTP^/Nap1_2_/H2A-H2B complex. Blob picking was used first and then the particles containing Kap114 and Ran^GTP^ were used for Topaz training. Topaz-picked particles were cleaned up and submitted for 3D reconstruction to obtain four maps. The population with density for H2A-H2B and Nap1_2_ (albeit poor density) was used for non-uniform (NU; gray map; EMD-44151) and local refinement (cyan map; EMD-44150) to obtain the final maps. The maps were overlayed onto the final Nap1_2_•H2A-H2B•Kap114•Ran^GTP^ structure (9B3I; in cyan•yellow-red•gray•green). Below is the consensus map colored by local resolution. **(F)** Phenix map-to-model FSC curves for the composite map (EMD-44141). **(G)** 3D angular distribution of the particles used for reconstruction. Left orientation is the same as in E. **(H)** Directional FSCs unmasked and masked by cryoSPARC refine mask, for the consensus map. Source data are available for this figure: SourceData FS2.

All importins bind Ran^GTP^ tightly, usually causing importin•cargo dissociation to release cargo in the nucleus (Dasso, 2002; Görlich et al., 1996; Hahn and Schlenstedt, 2011). However, a few exceptions have been reported, such as the very tight-binding TBP, which is released from Kap114 only at its target gene promoters (Liao et al., 2023; Pemberton et al., 1999). The Kap114/IMP9-H2A-H2B interaction is another exception as Ran^GTP^ binds Kap114/IMP9•H2A-H2B to form a stable ternary Ran^GTP^•Kap114/IMP9•H2A-H2B complex (Fig. 4 A) (Jiou et al., 2023; Shaffer et al., 2023). Similarly, Kap114-Nap1 interaction persists in the presence of Ran^GTP^ as shown by pull-down assays and AUC analysis (Fig. 1 B and Fig. S2, A–C). SEC-MALS analysis showed that an equimolar mix of Kap114, Nap1_2_, H2A-H2B, and Ran^GTP^ produced a major peak consistent with a complex that contains one molecule of each of the four proteins (Fig. 1 C).

### Structure determination of the Kap114/Nap1_2_/H2A-H2B/Ran^GTP^ complex

**Figure 2.**
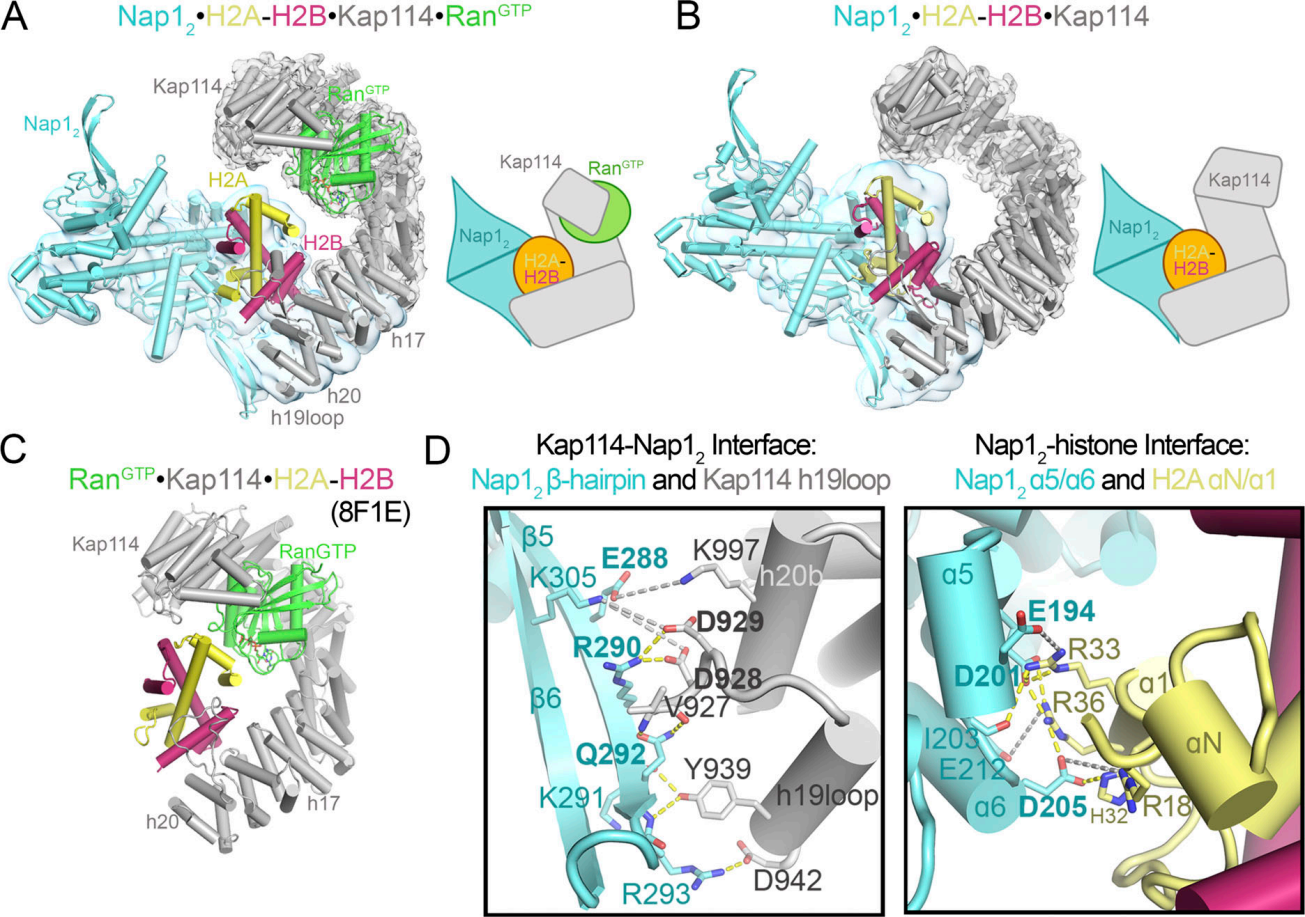
**Structure of the Nap1**_**2**_**•H2A-H2B•Kap114•Ran**^**GTP**^ **complex. (A)** The Nap1_2_•H2A-H2B•Kap114•Ran^GTP^ structure, with the consensus/local refined maps (gray/cyan) overlayed and a cartoon schematic on the right. **(B)** The Nap1_2_•H2A-H2B•Kap114 structure shown is as in A. **(C)** The Ran^GTP^•Kap114•H2A-H2B structure (8F1E). **(D)** Kap114-Nap1_2_ contacts (left) and H2A-H2B-Nap1_2_ contacts (right) in the Nap1_2_•H2A-H2B•Kap114 structure. Dashed lines show intermolecular contacts <4.0 Å (yellow) and <8.0 Å (light gray). See more in Figs. S2, S3, and S4.

**Figure S3.**
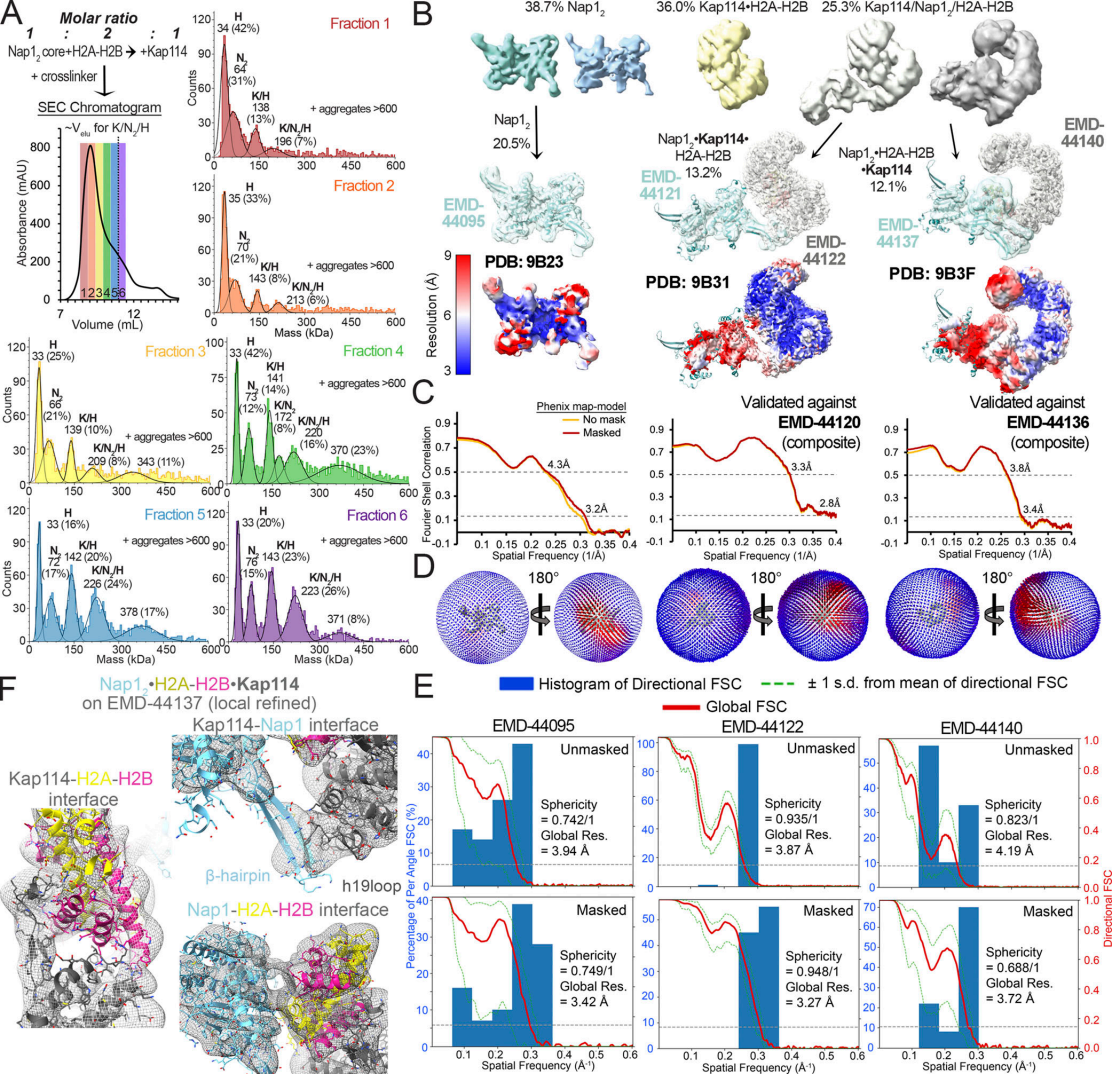
**Cryo-EM analysis of the Kap114/Nap1**_**2**_**/H2A-H2B ternary complexes. (A)** Top left: Schematic of how the cryoEM sample was assembled with its SEC chromatogram below, with fractions 1–6 colored red to purple. The typical elution volume of an uncrosslinked complex of Kap114, Nap1_2_, and H2A-H2B is ∼11 ml (dotted line). Each fraction was analyzed by mass photometry, and the data are plotted as in Fig. S2 D. Protein aggregates beyond 600 kDa were not displayed. Proteins in the ∼340–380 kDa peak may be crosslinked complexes of K/N_2_/H with K/H; such a large complex was not observed in AUC or SEC-MALS studies. Fraction 6, most enriched with the 1:1:1 Kap114:Nap1_2_:H2A-H2B complex and has the least large aggregates, was used for cryo-EM grid preparation. **(B)** Distribution of the ∼1 million particles produced by the cryo-EM data: 38.7% are Nap1_2_, 36.0% Kap114•H2A-H2B, and 25.3% ternary complex of Kap114/Nap1_2_/H2A-H2B (see more statistics in Table S1). The small population of ternary complex is likely due to destabilization of the assembly by excess H2A-H2B as shown in Fig. S1. Ternary complex particles were classified into two evenly divided classes that produced two high-resolution maps and structures. Left to right: Final consensus maps for Nap1_2_ (EMD-44095; cyan) overlayed with the final model (9B23; cyan), consensus (EMD-44122; gray) and local refined (EMD-44121; cyan) maps for Nap1_2_•Kap114•H2A-H2B overlayed with the final model (9B31; cyan•gray•yellow-red), and consensus (EMD-44140; gray) and local refined (EMD-44137; cyan) maps for Nap1_2_•H2A-H2B•Kap114 overlayed with the final model (9B3F; cyan•yellow-red•gray). Consensus maps are also shown below colored by local resolution. Nap1_2_•Kap114•H2A-H2B resembles the Kap114•H2A-H2B structure (8F0X), with Nap1_2_ contacting Kap114 h19loop via the β-hairpin. This structure may represent a ternary complex that is falling apart or it may be one configuration of a dynamic ternary complex ensemble. **(C)** Left to right: Phenix map-to-model FSC curves for the consensus map for Nap1_2_, or the composite maps for Nap1_2_•Kap114•H2A-H2B (EMD-44120) and Nap1_2_•H2A-H2B•Kap114 (EMD-44136). **(D)** 3D angular distribution of the particles that were used for reconstructions of the consensus maps above. The left orientation is same as in B. **(E)** Directional FSCs unmasked and masked by cryoSPARC refine mask of the consensus maps above. **(F)** The Nap1_2_•H2A-H2B•Kap114 structure overlayed onto the local refined map (gray mesh), zoomed into the Kap114-H2A-H2B, Kap114-Nap1_2_ and Nap1_2_-H2A-H2B interfaces.

We assembled the quaternary Kap114/Nap1_2_/H2A-H2B/Ran^GTP^ complex and solved the cryo-EM structure to 2.9 Å resolution (Table 1; Fig. 2 A; and Fig. S2, E–H) (Fung et al., 2024a, 2024b, 2024c). For comparison, we also performed cryo-EM analysis with a mixture of Kap114, Nap1_2_, and excess H2A-H2B without Ran^GTP^, which produced a heterogenous mixture of particles that included unliganded Nap1_2_, the binary Kap114•H2A-H2B complex, and small populations of two different assemblies containing Kap114, Nap1_2_, and H2A-H2B (Fig. 2 B; Table S1; and Fig. S3). Excess histone in the sample had destabilized the Kap114/Nap1_2_/H2A-H2B ternary complex (more discussion in Fig. S1). Structures of the two ternary Kap114/Nap1_2_/H2A-H2B complexes are shown in Fig. 2 B and Fig. S3, for comparison with the quaternary Kap114/Nap1_2_/H2A-H2B/Ran^GTP^ complex (additional discussion in Fig. S3 legend) (Jiou et al., 2024a, 2024b, 2024c).

**Table 1.** Cryo-EM data collection, refinement, and validation statistics

	Nap1_2_•H2A-H2B•Kap114•Ran^GTP^			Nap1_2_•H2A-H2B•Kap114		
	Consensus map EMD-44151	Locally refined map for Nap1_2_•H2A-H2B EMD-44150	Composite map PDB: 9B3I EMD-44141	Consensus map EMD-44140	Locally refined map for Nap1_2_ •H2A-H2B EMD-44137	Composite map PDB: 9B3F EMD-44136
Data collection and processing						
Magnification (X)	165,000			105,000		
Voltage (kV)	300			300		
Electron exposure (e^−^/Å^2^)	50			52		
Defocus range (μm)	0.9–2.2			1.5–2.5		
Pixel size (Å)	0.738			0.83		
Symmetry imposed	C1			C1		
Initial particle images (no.)	1,381,753			4,314,112		
Final particle images (no.)	133,516			113,011		
Map resolution (Å)	2.88	3.97		3.54	5.62	
FSC threshold	0.143					
Refinement						
Initial model used (PDB code)			8F1E, 9B3F			AF-P53067-F1, 8F1E, 9B23
Model composition						
Non-hydrogen atoms			14,951			13,451
Protein residues			1,854			1,668
Mean *B* factors (Å^2^)						
Protein/Ligand			112.36/40.96			317.38
R.m.s. deviations						
Bond lengths (Å)			0.006			0.002
Bond angles (°)			0.781			0.505
CCvolume/mask			0.74/0.73			0.70/0.70
Validation						
MolProbity score			1.55			1.46
Clashscore			10.80			7.64
Poor rotamers (%)			0.24			0
Ramachandran plot						
Favored (%)			98.20			97.82
Allowed (%)			1.80			2.18
Disallowed (%)			0			0
CaBLAM outliers (%)			0.82			1.04
EMRinger score			2.45			0.94

The final cryo-EM map of the Kap114/Nap1_2_/H2A-H2B/Ran^GTP^ complex is well-defined for Kap114 and Ran^GTP^ but the local resolution and map feature for Nap1_2_ and H2A-H2B are poor. Local refinement produced a 4.0 Å map with improved map features. We built the structure of the quaternary complex using initial models from two other structures: (1) Ran^GTP^ bound to the N-terminal HEAT repeats of Kap114 from the Ran^GTP^•Kap114•H2A-H2B structure (PDB: 8F1E, Fig. 2 C) (Jiou et al., 2023) and (2) Kap114 repeats h17-h20 bound to Nap1_2_ and H2A-H2B from the Nap1_2_•H2A-H2B•Kap114 structure obtained in this study (Fig. 2 B). We named the quaternary assembly the Nap1_2_•H2A-H2B•Kap114•Ran^GTP^ complex (Fig. 2 A).

**Figure S4.**
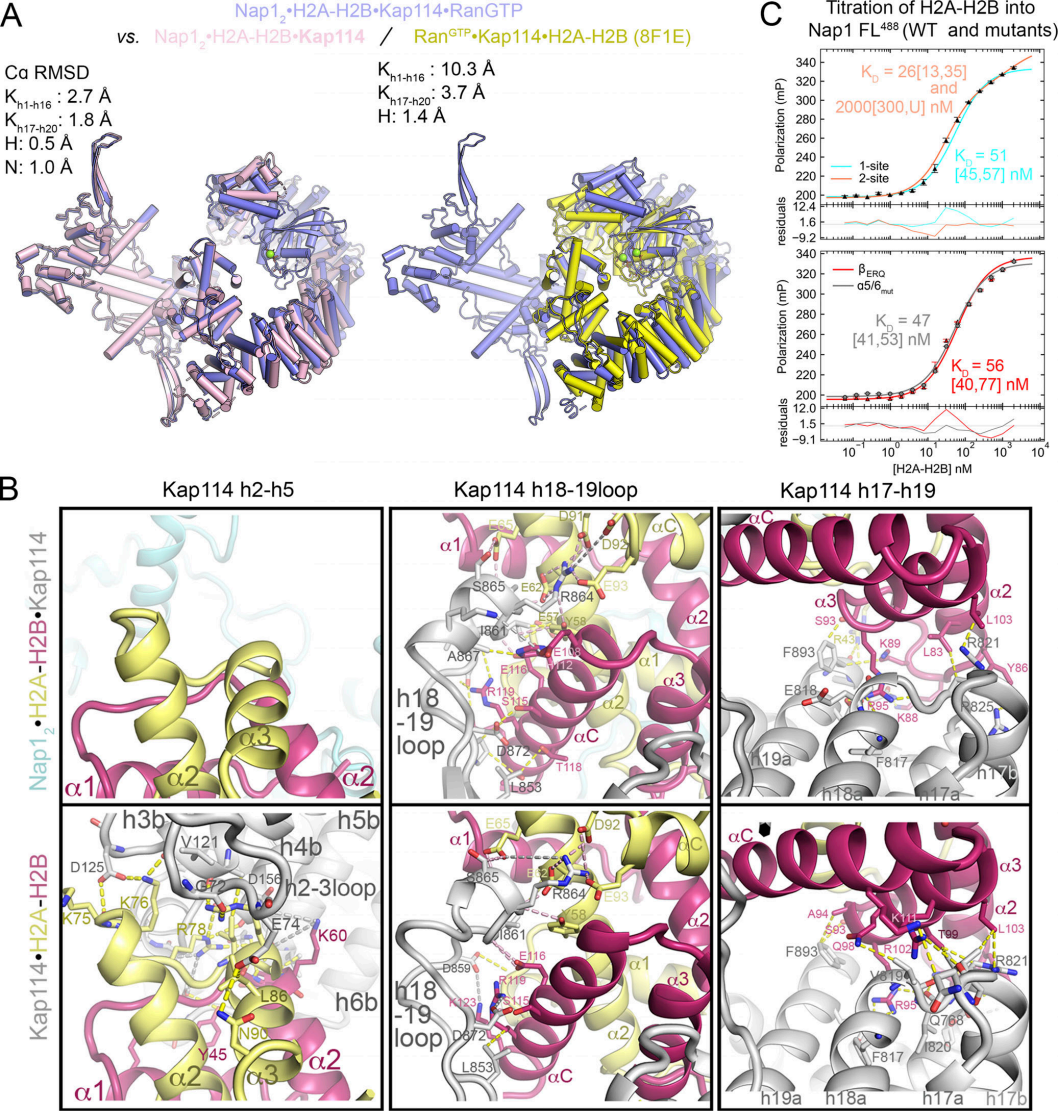
**Structural comparisons of Nap1**_**2**_**•H2A-H2B•Kap114•Ran**^**GTP**^ **with other structures. (A)** The H2A-H2B heterodimers of Nap1_2_•H2A-H2B•Kap114•Ran^GTP^ (purple), Nap1_2_•H2A-H2B•Kap114 (pink; left) and Ran^GTP^•Kap114•H2A-H2B (yellow; right, PDB: 8F1E) were aligned. The Cα r.m.s.d. values of different molecules, calculated in PyMOL without realignment, are reported. **(B)** Left to right: Interactions of H2A-H2B with HEAT repeats 2–5 of Kap114 or lack thereof, and the persistent interactions with h18-19loop of Kap114 and h17-h19 in Nap1_2_•H2A-H2B•Kap114 and Kap114•H2A-H2B (PDB: 8F0X). **(C)** Nap1 mutations at its interfaces with Kap114 or H2A-H2B in Nap1_2_•H2A-H2B•Kap114 do not affect H2A-H2B binding as seen in fluorescence polarization assay using 10 nM Nap1_2_ FL labeled with XFD488 (Nap1 FL^488^). Data points are averages ± SD. of triplicate measurement. Top: WT Nap1 tiration. The lines show data fitted with one- or two-site binding and residuals are plotted below. Dissociation constants are recorded with the 95% confidence interval obtained by error-surface projection method in brackets. The data is better fitted with two-site binding, which is consistent with previous works by Ohtomo et al. that reported human Nap1_2_ binding two copies of H2A-H2B, one bound to the C-terminal acidic tails and one to the core. Bottom: Nap1 mutants β_ERQ_ and α5/6_mut_. One-site binding model was used for fitting as data could not be fitted confidently with two-site binding. All Nap1 mutants bound H2A-H2B with high affinity in the low nM range.

Nap1_2_•H2A-H2B•Kap114•Ran^GTP^ resembles one of the ternary complexes, the Nap1_2_•H2A-H2B•Kap114 complex, except that no Ran^GTP^ is bound in the latter (Fig. 2, A and B). In both complexes, H2A-H2B is sandwiched between the C-terminal HEAT repeats of Kap114 and one subunit of the Nap1_2_ (Fig. 2, A and B). In the quaternary Nap1_2_•H2A-H2B•Kap114•Ran^GTP^ complex, Ran^GTP^ binds to HEAT repeats h1–4, 8, and 12–14 of Kap114 in the same way as in the Ran^GTP^•Kap114•H2A-H2B structure (Fig. 2, A and C). Comparison of the ternary and quaternary complexes shows that Ran-binding stabilizes a Kap114 conformation with an N-terminus closer to the bound H2A-H2B, but not as close as in the Ran^GTP^•Kap114•H2A-H2B structure where Nap1_2_ is absent (Fig. S4 A).

### The Nap1_2_ β-hairpin and Kap114 h19loop are binding hotspots

In both the quaternary Nap1_2_•H2A-H2B•Kap114•Ran^GTP^ and ternary Nap1_2_•H2A-H2B•Kap114 complexes, H2A-H2B interacts with the C-terminal HEAT repeats of Kap114 just like in other H2A-H2B-bound Kap114 structures, covering an extensive surface area of ∼1,600 Å^2^ (Fig. S4 B). The smaller (∼120 Å^2^) Kap114-Nap1_2_ interface in Nap1_2_•H2A-H2B•Kap114•Ran^GTP^ (and in Nap1_2_•H2A-H2B•Kap114) involves the β-hairpin of one Nap1_2_ subunit (residues E288, R290 and Q292) contacting the Kap114 h19loop and the h20b helix (Fig. 2 D, left). The Nap1_2_–histone interface involves acidic residues of the α4–6 helices of one Nap1_2_ subunit contacting basic residues of the H2A αN and α1 helix (Fig. 2 D, right).

**Figure 3.**
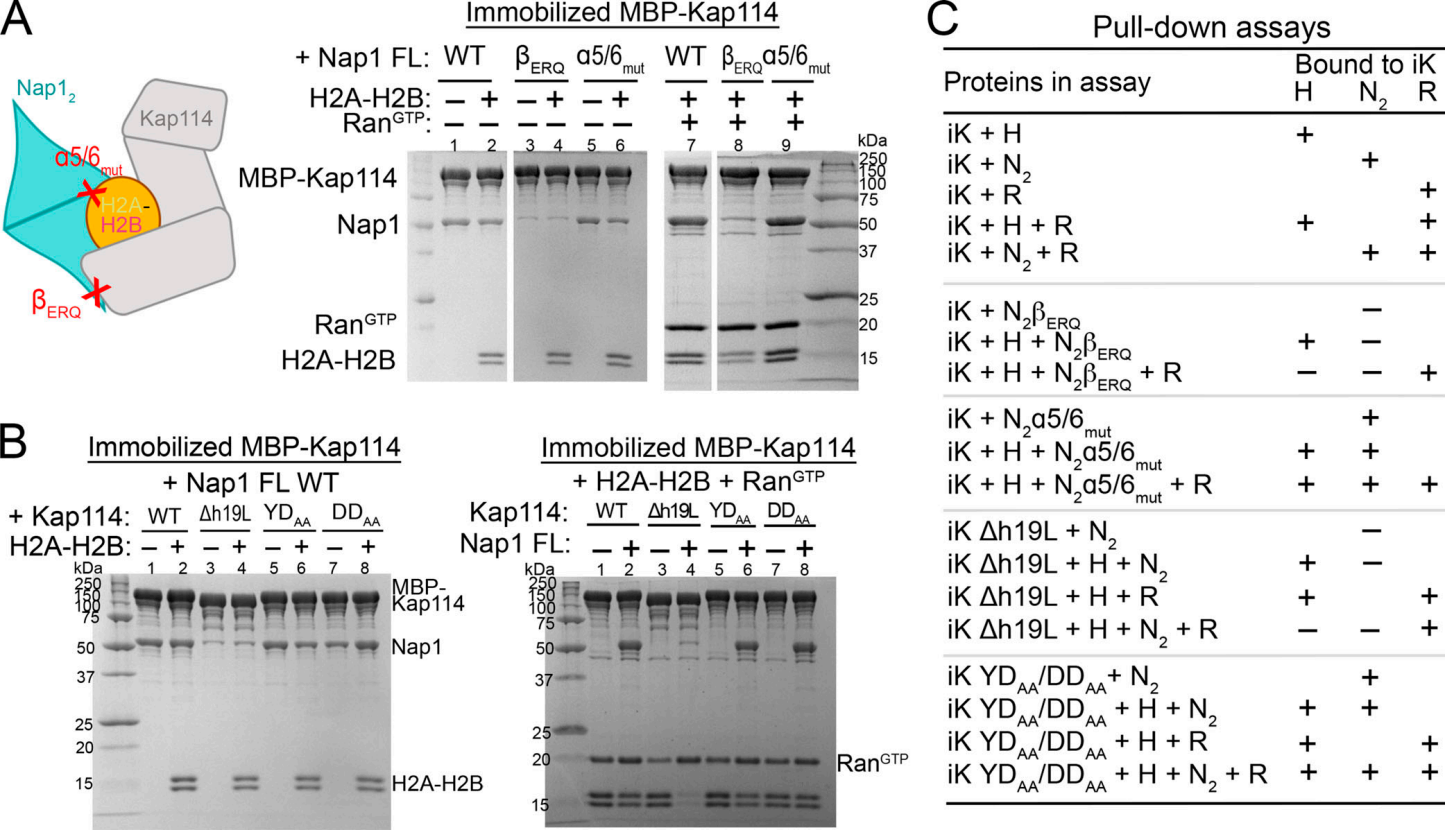
**The Nap1**_**2**_ **β-hairpin and Kap114 h19loop are binding hotspots. (A)** Pull-down assay with equimolar MBP-Kap114 (1 µM), Nap1_2_ (WT, β-hairpin mutant β_ERQ_ (E288A/R290A/Q292A) or histone-binding site mutant α5/6_mut_ (E194A/D201A/D205A), as indicated by schematic on the left) ± H2A-H2B ± Ran^GTP^. Bounds proteins were visualized by Coomassie-stained SDS-PAGE. Controls in Fig. S4 C. **(B)** Kap114 h19loop mutants Δh19L (h19loop deleted), YD_AA_ (Y939A/D942A), and DD_AA_ (D928A/D929A) in pull-down assay as in A. **(C)** Summary of pull-down assays with immobilized MBP-Kap114 (iK). Source data are available for this figure: SourceData F3.

We mutated Nap1 residues that participate in Kap114-Nap1_2_ and Nap1_2_–histone interactions to assess their importance in forming quaternary and ternary complexes (Fig. 3 A, controls in Fig. S4 C). Alanine mutations of the Nap1_2_ β-hairpin residues E288, R290, and Q292 (mutant β_ERQ_) that contact Kap114 abolished Nap1_2_ pull-down by MBP-Kap114 in the absence and presence of H2A-H2B and Ran^GTP^. However, mutations of Nap1_2_ residues E194, D201, and D205 (mutant α5/6_mut_) that contact H2A-H2B did not affect ternary or quaternary complex formation. These results support the importance of the Nap1_2_ β-hairpin for Kap114-binding, in contrast to Nap1_2_-H2A-H2B interactions, which are less important for ternary and quaternary complex formation.

We also mutated contact residues in Kap114 (Fig. 3 B). The Kap114 h19loop binds the Nap1_2_ β-hairpin and it is therefore not surprising that truncating the loop (Kap114 Δh19L) abolished Kap114-Nap1 pull-down and formation of the ternary and quaternary complexes. However, mutating just D928 and D929 or Y939 and D942 (mutant Kap114 DD_AA_ and YD_AA_) that contact Nap1_2_ in the Nap1_2_•H2A-H2B•Kap114•Ran^GTP^ and Nap1_2_•H2A-H2B•Kap114 structures did not affect Kap114-Nap1 pull-down. There are nearby acidic/electronegative Kap114 side chains that may also interact with the Nap1_2_ β-hairpin (Fig. 2 D).

In summary, mutagenesis results indicate that although the interfaces between the Nap1_2_ β-hairpin and Kap114 h19loop in both the ternary and quaternary structures are relatively small, these interactions are essential for binding (Fig. 3 C). Therefore, these results validate the cryo-EM structures of both Nap1_2_•H2A-H2B•Kap114•Ran^GTP^ and Nap1_2_•H2A-H2B•Kap114 complexes. Our structural and biochemical results are also consistent with previously published results that suggested the importance of the Nap1 β-hairpin for nuclear localization of Nap1 in the yeast nuclei (Hodges et al., 2005; Mosammaparast et al., 2002, 2005).

### Kap114 and Nap1_2_: Co-chaperoning H2A-H2B in the absence of Ran^GTP^

**Figure 4.**
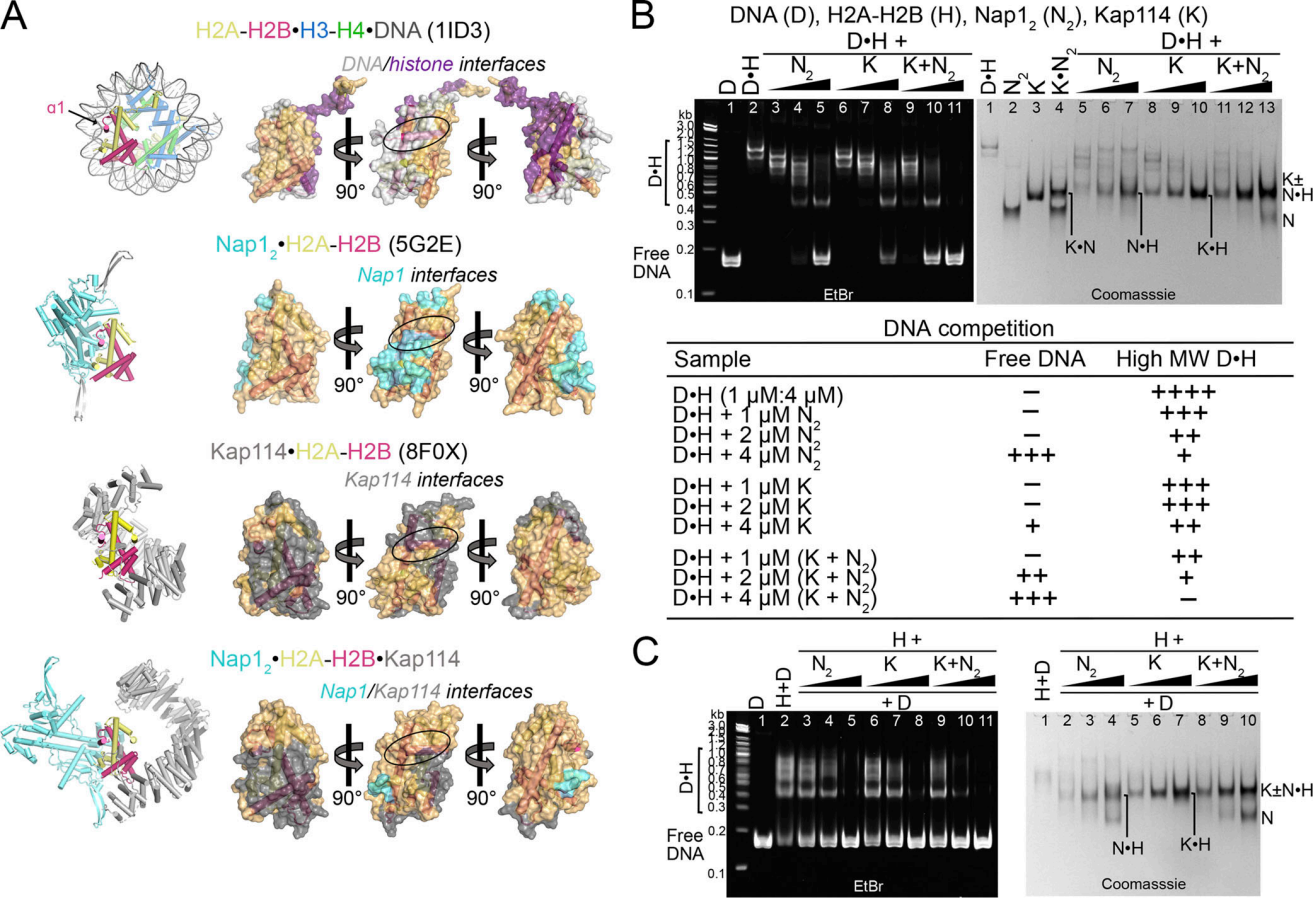
**Kap114 and Nap1**_**2**_ **co-chaperone H2A-H2B in the absence of Ran**^**GTP**^**. (A)** Left, top to bottom: Structures of the nucleosome (1ID3), Nap1_2_•H2A-H2B (5G2E), Kap114•H2A-H2B (8F0X), and Nap1_2_•H2A-H2B•Kap114. Right panels, top to bottom: three views of the semi-transparent H2A-H2B surface with cartoon representation underneath, for the corresponding structures in the left panel. Binding interfaces (PDBePISA) are colored according to binding partners. Ovals highlight the H2B α1 helix, which is buried by Nap1_2_ in Nap1_2_•H2A-H2B and by Kap114 in Kap114•H2A-H2B, and very likely restricted in access by Nap1_2_ in Nap1_2_•H2A-H2B•Kap114. **(B)** DNA competition assays of DNA•H2A-H2B complex (D•H) added to Nap1 (N_2_), Kap114 (K), or Kap114+Nap1_2_ mixture (K+N_2_), in the absence of Ran^GTP^. Summary of DNA competition assay results as shown below. Samples were visualized using ethidium bromide- and Coomassie-stained native PAGE gels. **(C)** Similar assay as in B, but all proteins were assembled in complexes before the addition of DNA. Source data are available for this figure: SourceData F4.

Nap1 and Kap114/*Hs* IMP9 had previously been identified as highly effective histone chaperones that prevent aggregation of H2A-H2B with DNA (Chang et al., 1997; Chen et al., 2016; Huang et al., 2020; Jiou et al., 2023; Mosammaparast et al., 2002; Padavannil et al., 2019). Consistent with their histone chaperone function, the Nap1_2_ and Kap114 proteins in Nap1_2_•H2A-H2B•Kap114 shield substantial portions (∼1,800 Å^2^) of the nucleosomal H3-H4 and DNA binding regions of H2A-H2B (Fig. 4 A). We performed DNA competition assays to probe the H2A-H2B chaperoning activity of Kap114 and Nap1_2_, individually and together, within an equimolar ternary complex.

In this assay, H2A-H2B chaperoning would result in the disappearance of low-mobility DNA•H2A-H2B bands and the appearance of high-mobility–free DNA bands. We performed the experiments by either assembling DNA•H2A-H2B first and then titrating in Nap1_2_ and/or Kap114 (Fig. 4 B) or assembling complexes of H2A-H2B with Kap114 and/or Nap1_2_ first before titrating them into DNA (Fig. 4 C). To ensure a stable and intact ternary complex, we added Kap114 and/or Nap1_2_ to DNA•H2A-H2B in an equimolar ratio, such as in lanes 5, 8, and 11 in EtBr gels (left) of Fig. 4, B and C, to avoid excess histone, which we know destabilizes the ternary complex (Fig. S1). Regardless of the order of addition, Kap114 and Nap1_2_ together chaperone H2A-H2B from aggregation with DNA more effectively than either Kap114 or Nap1_2_ alone.

Both Nap1 and Kap114 individually chaperoned H2A-H2B, decreasing low-mobility DNA•H2A-H2B bands and producing free DNA bands (Fig. 4 B). Interestingly, Nap1_2_ is a more effective chaperone than Kap114 (Fig. 4 B, left gel: lanes 3–5 versus 6–8) even though the Nap1_2_•H2A-H2B interface (∼800 Å^2^) is smaller than the Kap114•H2A-H2B interface (∼2,200 Å^2^). It is possible that dynamic interactions of the Nap1_2_ C-terminal tail (not present in the Nap1_2_•H2A-H2B structure) and Nap1_2_ oligomerization with H2A-H2B may provide additional shielding of H2A-H2B (Aguilar-Gurrieri et al., 2016; Ohtomo et al., 2023).

Most importantly, the presence of both Kap114 and Nap1_2_ enhances the chaperoning of H2A-H2B compared to when either is present alone (Fig. 4 B, left gel: compare lanes 5, 8, versus 11). This enhanced chaperoning occurs despite less histone surface being shielded in the Nap1_2_•H2A-H2B•Kap114 complex (∼1,600 Å^2^) compared to the binary Kap114•H2A-H2B structure (∼2,200 Å^2^). We propose that additional surfaces of the histone, when bound to both Kap114 and Nap1_2_, become inaccessible beyond the interfaces depicted in Fig. 4 A due to steric hindrance. For instance, although Nap1_2_ in Nap1_2_•H2A-H2B•Kap114 does not form <4 Å contacts with the DNA-binding region at the H2B α1 helix, it is <10 Å away, close enough to likely restrict access of other macromolecules to the histone (Fig. 4 A). Furthermore, the ternary complex is dynamic and the Nap1_2_•H2A-H2B•Kap114 structure represents only one configuration of an ensemble of possible conformations (Fig. S3 B).

In summary, the structural and biochemical results above support Kap114 and Nap1_2_ in the Nap1_2_•H2A-H2B•Kap114 ternary complex cooperating to effectively co-chaperone H2A-H2B.

### Kap114/Nap1 co-chaperoning and nucleosome assembly in the presence of Ran^GTP^

**Figure 5.**
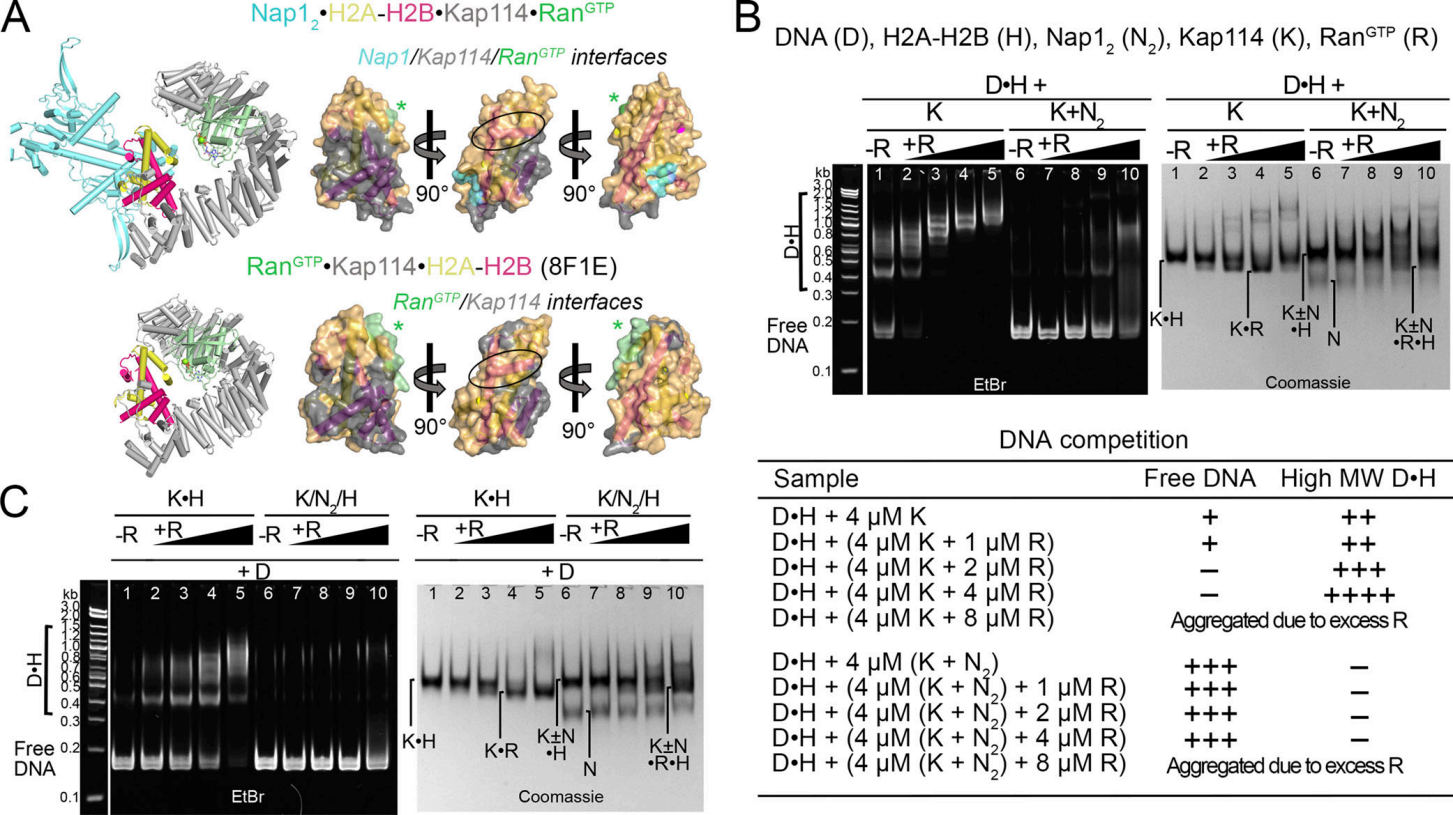
**Kap114 and Nap1**_**2**_ **co-chaperone H2A-H2B in the presence of Ran**^**GTP**^**. (A)** Depicted as in Fig. 4 A, histone interaction interfaces in Nap1_2_•H2A-H2B•Kap114•Ran^GTP^ and Ran^GTP^•Kap114•H2A-H2B (8F1E). The green asterisks (*) indicate transient Ran^GTP^-H2A-H2B contacts. H2B α1 (black oval) is likely inaccessible due to the proximity of Nap1_2_ in Nap1_2_•H2A-H2B•Kap114•Ran^GTP^ just like Nap1_2_•H2A-H2B•Kap114; however, it is exposed in Ran^GTP^•Kap114•H2A-H2B, likely explaining why histone is not effectively chaperoned in this complex. **(B)** DNA competition assays of DNA•H2A-H2B complex (D•H; 1 µM:4 µM) added to Kap114 (K) or Kap114+Nap1_2_ mixture (K+N_2_), with a titration of Ran^GTP^ (concentrations indicated in the table of summary of results below. Samples were visualized using ethidium bromide- and Coomassie-stained native PAGE gels. **(C)** Similar assay as in B, but all proteins were assembled in complexes before the addition of DNA. Source data are available for this figure: SourceData F5.

The nuclear/quaternary Nap1_2_•H2A-H2B•Kap114•Ran^GTP^ complex buries ∼1,900 Å^2^ of the H2A-H2B surface. Within this complex, Nap1_2_ and Kap114 collectively shield the DNA binding interfaces of H2A-H2B, mirroring the arrangement observed in the cytosolic/ternary Nap1_2_•H2A-H2B•Kap114 structure (Fig. 5 A). The similarity in protein arrangements between the ternary and quaternary complexes suggests that, even in the presence of Ran^GTP^, Kap114 and Nap1_2_ likely continue to prevent H2A-H2B from aggregating with DNA. To test this hypothesis, we conducted DNA competition assays using Kap114, H2A-H2B, with and without Nap1_2_ and titrated in Ran^GTP^ (Fig. 5, B and C).

In the absence of Ran^GTP^, Kap114 alone can chaperone H2A-H2B. However, its efficacy as a histone chaperone decreases substantially in the presence of the GTPase (Fig. 5 B, compare lane 1 with lanes 2–5). Interestingly, when both Nap1_2_ and Kap114 are present, H2A-H2B chaperoning remains unaffected by the presence of Ran^GTP^ (Fig. 5, compare lane 6 with lanes 7–10). This indicates that the histone is chaperoned effectively by Kap114 and Nap1_2_ within the quaternary/nuclear Nap1_2_•H2A-H2B•Kap114•Ran^GTP^ complex.

**Figure 6.**
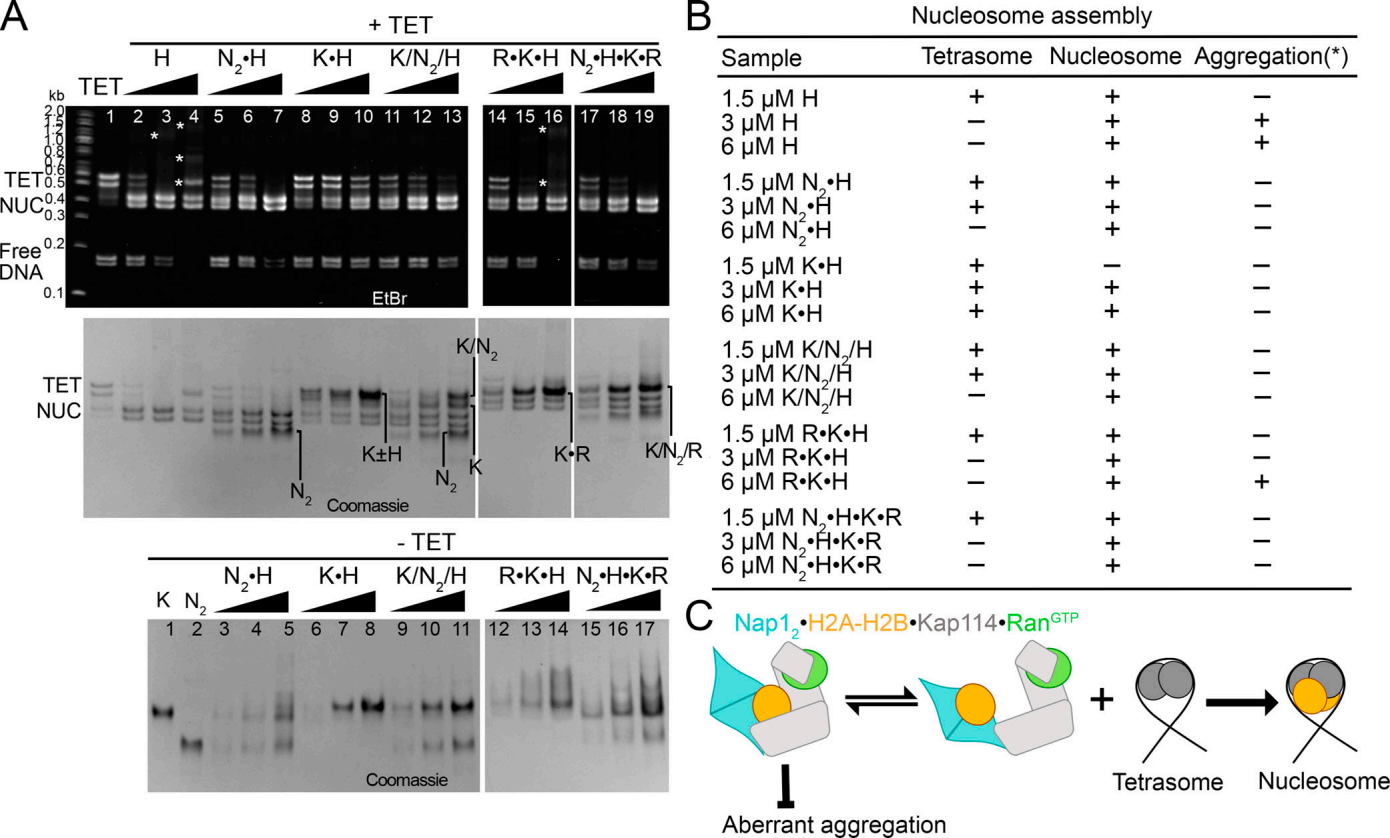
**Nap1**_**2**_**•H2A-H2B•Kap114•Ran**^**GTP**^ **effectively deposits H2A-H2B on tetrasomes. (A)** Nucleosome assembly assay: H2A-H2B (H) pre-mixed with increasing concentrations of Nap1_2_ (N_2_) and/or Kap114 (K), without and with Ran^GTP^ (R), before the addition of ∼3–4 µM tetrasomes (TET; see Materials and methods). Samples were visualized using ethidium bromide- (top) and Coomassie-stained (middle) native PAGE gels. Nap1_2_ allows nucleosome (NUC) formation while Kap114 inhibits it. Both Ran^GTP^•Kap114•H2A-H2B and Nap1_2_•H2A-H2B•Kap114•Ran^GTP^ form NUC effectively, but the former do not shield H2A-H2B from aggregation with DNA (white asterisks*). Bottom gel: control samples were without TET, where increasing the protein concentration did not affect mobility, indicating that protein complexes were stably formed at 1.5 µM. **(B)** Summary of protein concentrations and nucleosome assembly results. **(C)** Hypothetical model of how Nap1_2_•H2A-H2B•Kap114•Ran^GTP^ promotes H2A-H2B transfer from Kap114 to assembling nucleosomes. The quaternary complex remains an effective chaperone of H2A-H2B and shields the bound histone from aberrant aggregation while the Kap114-bound Ran^GTP^ likely promotes histone release to the Kap114-bound Nap1_2_, which can then effectively transfer H2A-H2B to tetrasomes to form nucleosomes. Source data are available for this figure: SourceData F6.

We also performed nucleosome assembly assays to assess the impact of Kap114, Nap1_2_, and Ran^GTP^ on H2A-H2B deposition into tetrasomes, leading to nucleosome formation (Fig. 6, A and B). The results corroborate previous findings that Nap1_2_ allows H2A-H2B deposition into nucleosomes (Fig. 6 A, lanes 5–7) (Andrews et al., 2010). In contrast, Kap114 inhibits H2A-H2B deposition into nucleosomes (Fig. 6 A, lanes 8–10) (Jiou et al., 2023). When both Nap1_2_ and Kap114 are present, as in the ternary Nap1_2_•H2A-H2B•Kap114 complex, H2A-H2B can still be deposited into nucleosomes, like with Nap1_2_ alone (Fig. 6 A, compare lanes 11–13 with 5–7). In the presence of both Ran^GTP^ and Kap114, as in the Ran^GTP^•Kap114•H2A-H2B complex, the GTPase facilitates histone release from Kap114, thus promoting nucleosome formation (Fig. 6 A, lanes 14–16). However, Ran^GTP^•Kap114•H2A-H2B does not effectively shield H2A-H2B from aggregation with DNA, consistent with the low mobility DNA•H2A-H2B bands and smear marked with white asterisks in lane 16 of Fig. 6 A. When Nap1_2_, Kap114 and Ran^GTP^ are all present, as in the quaternary Nap1_2_•H2A-H2B•Kap114•Ran^GTP^ complex, H2A-H2B deposition is more effective than either Nap1_2_ alone or the ternary Nap1_2_•H2A-H2B•Kap114 complex (compare lanes 6, 12 and 18 of Fig. 6 A).

In summary, Kap114 and Nap1_2_ in the quaternary Nap1_2_•H2A-H2B•Kap114•Ran^GTP^ complex cooperate to chaperone H2A-H2B. Without Nap1_2_, Ran^GTP^ would bind Kap114•H2A-H2B, reorienting the bound histone and increasing its exposure to DNA and non-specific interactions. However, Nap1_2_ binding H2A-H2B alongside Kap114 in the nuclear/quaternary Nap1_2_•H2A-H2B•Kap114•Ran^GTP^ complex shields the histone from aberrant aggregation with DNA while Nap1_2_ and Ran^GTP^ work together to facilitate specific release of the histone from Kap114 to the tetrasome.

### H2A-H2B transfer from Nap1_2_•H2A-H2B•Kap114•Ran^GTP^ to nucleosome

To explore how Kap114, Nap1_2_, and Ran^GTP^ cooperate in transferring H2A-H2B onto assembling nucleosomes, we used a Kap114 mutant that cannot bind Nap1_2_. We performed pull-down binding assays with equimolar amounts of Kap114 Δh19L, Nap1_2_, H2A-H2B, and Ran^GTP^ (Fig. 3 B). Despite its inability to bind Nap1_2_, the Kap114 Δh19L mutant successfully captured H2A-H2B in the presence of Nap1_2_, forming a Kap114 Δh19L•H2A-H2B complex, consistent with H2A-H2B’s high affinity for the importin (Fig. 3 B) (Jiou et al., 2023). Additionally, Kap114 Δh19L also pulled down H2A-H2B in the presence of Ran^GTP^, forming the Ran^GTP^•Kap114 Δh19L•H2A-H2B complex (Fig. 3 B). However, when both Ran^GTP^ and Nap1_2_ were present, Kap114 Δh19L failed to capture H2A-H2B, suggesting that Ran^GTP^ enabled free Nap1, which cannot bind Kap114 Δh19L, to displace the histone from the importin. These findings imply that in the Nap1_2_•H2A-H2B•Kap114•Ran^GTP^ complex, Ran^GTP^ may facilitate the transfer of H2A-H2B from Kap114 to Nap1_2_, optimizing its deposition into nucleosomes (Fig. 6 C). This quaternary assembly likely provides a targeted pathway to efficiently transfer H2A-H2B from Kap114 to Nap1_2_ to the tetrasome, while minimizing the risk of non-specific or aberrant interactions in the nucleus.

## Discussion

We have revealed the structure of the Nap1_2_•H2A-H2B•Kap114•Ran^GTP^ quaternary complex, which represents the nuclear state of the co-import complex of Nap1_2_ and H2A-H2B with their importer Kap114. A similar structural arrangement of the ternary Nap1_2_•H2A-H2B•Kap114 complex suggests that it is the cytoplasmic import complex. In both structures, the DNA-binding surfaces of H2A-H2B are shielded by either Kap114 or Nap1_2_, consistent with Kap114 and Nap1_2_ co-chaperoning H2A-H2B, preventing aberrant interactions. Nap1_2_ in the quaternary/nuclear Nap1_2_•H2A-H2B•Kap114•Ran^GTP^ complex is essential for chaperoning H2A-H2B in the presence of Ran^GTP^. The assembly efficiently deposits H2A-H2B into nucleosomes.

Nap1 is mostly cytoplasmic at a steady state (Kellogg et al., 1995), but the Pemberton group showed that it shuttles between the nucleus and the cytoplasm (Mosammaparast et al., 2002). They also identified Nap1 as a cofactor in Kap114-mediated nuclear import of H2A-H2B and revealed its roles in chromatin assembly, transcription elongation, and mRNP biogenesis within the nucleus, that others also reported (Aguilar-Gurrieri et al., 2016; Altman and Kellogg, 1997; Andrews et al., 2010; Chen et al., 2016; Del Rosario and Pemberton, 2008; Dronamraju et al., 2017; Hsu et al., 2019; Kellogg and Murray, 1995; Krajewski, 2020; Levchenko and Jackson, 2004; Mazurkiewicz et al., 2006; Moshkin et al., 2013; Nagae et al., 2023; Park et al., 2005; Sharma and Nyborg, 2008; Vlijm et al., 2012). In addition to its well-established role in nucleosome assembly/remodeling, Nap1 also participates in DNA repair (Fan et al., 2024; Gao et al., 2012; Liu et al., 2009; Machida et al., 2014). The Pemberton group further demonstrated direct interactions between Nap1, Kap114, H2A, and H2B, both in the absence and presence of Ran^GTP^ (Mosammaparast et al., 2002). Over two decades later, we now explain these interactions in the context of nuclear import, histone chaperoning, and nucleosome assembly.

Kap114/IMP9 binds H2A-H2B with extremely high affinity in the sub-nanomolar range (Jiou et al., 2023; Shaffer et al., 2023). This strong interaction aligns with the inhibitory effect of Kap114 on nucleosome assembly, as demonstrated here in Fig. 6 and our previous work (Jiou et al., 2023). In the nuclear Nap1_2_•H2A-H2B•Kap114•Ran^GTP^ complex, the presence of Nap1_2_ and Ran^GTP^ modulates H2A-H2B affinity, facilitating histone transfer to the assembling nucleosome or another histone chaperone. This targeted release of nuclear import cargo from Kap114 was previously suggested by Pemberton and colleagues who observed that the Ran^GTP^-mediated release of another cargo, the transcription factor TATA-binding protein (TBP), from Kap114 is enhanced by the presence of TATA-containing double-stranded DNA (Pemberton et al., 1999). Similar to the very high-affinity Kap114-H2A-H2B interaction, Kap114 also binds TBP with a very tight K_D_ of 1 nM or less (Liao et al., 2020), necessitating both Ran^GTP^ and its nuclear target for efficient cargo release.

Although Ran^GTP^ cannot efficiently dissociate the Kap114-TBP complex, no ternary Ran^GTP^•Kap114•TBP complex has been reported (Liao et al., 2020; Pemberton et al., 1999). This contrasts with the stable Nap1_2_•H2A-H2B•Kap114•Ran^GTP^ complex characterized in this study, which can be explained by the distinct cargo binding modes of Kap114. TBP binds in a region separate from the H2A-H2B binding site, interacting with a contiguous surface on the Kap114 solenoid that spans repeat h9-h13, along with contributions from the h8loop and the h19loop—overlapping with the Ran^GTP^ binding site (Liao et al., 2023). In contrast, the H2A-H2B binding site largely does not overlap with that of Ran^GTP^, and conformational changes in the flexible Kap114 enable simultaneous binding of both the histone and GTPase.

The very tight Kap114–TBP interaction likely contributes to Kap114’s ability to regulate TBP-dependent transcription in yeast by sequestering the transcription factor away from promoters (Liao et al., 2023). Similarly, the unusually strong Kap114–H2A–H2B interaction and the stability of the Nap1_2_•H2A-H2B•Kap114•Ran^GTP^ complex suggest a potential nuclear role for Kap114. This raises the possibility that Kap114 is involved in chromatin dynamics beyond simply delivering H2A-H2B to assembling the nucleosome. Our study elucidates the latter process, explaining how Nap1_2_ acts as a co-factor in Kap114-mediated H2A-H2B nuclear import, demonstrating how the importin and histone chaperone function together as co-chaperones.

The collaborative action of Kap114, Nap1_2_, and Ran^GTP^ could enable precise, efficient, and seamless transfer of H2A-H2B into assembling nucleosomes. Whether such localized release in cells is a more general property of importins is tempting to speculate and has been proposed but the data demonstrating this activity for other importins has not proven conclusive (Lee and Aitchison, 1999). Nonetheless, in the case of Kap114, we have provided valuable mechanistic insight into how nuclear import is tightly coordinated with the targeted release of cargo at its specific nuclear destination to ensure efficiency and mitigate deleterious effects of non-productive interactions.

## Materials and methods

### Protein constructs, expression, and purification

Kap114 was previously cloned into vector pGEX-4T3 (Cytiva) and subcloned into pMalE (New England BioLabs) using Sal1 and Not1 cut sites. pGEX-4T3 was modified with a TEV cleavage site between the GST tag and Kap114 whereas pMalE was modified with a His_6_-tag immediately N-terminus of MBP and a TEV cleavage site after the MBP. Mutant proteins were generated by site-directed mutagenesis or blunt-end ligations with primers and oligos listed in Table S2.

MBP-Kap114 was expressed in BL21 Gold cells grown in LB media, and protein expression was induced with 0.5 mM IPTG for 17 h at 18°C. Cells were harvested by centrifugation at 4,000 *g* (Sorvall BP6) and resuspended in lysis buffer containing 50 mM HEPES 7.0, 150 mM NaCl, 10% (vol/vol) glycerol, 5 mM DTT, 1 mM benzamidine, 10 μg/ml leupeptin, and 50 μg/ml AEBSF, and frozen. Thawed bacteria cells were lyzed using Emulsiflex homogenizer, the lysate was clarified by centrifugation at 48,400 *g* for 40 min at 4°C (Sorvall RC6), and the supernatant was added to amylose beads (New England Biolabs). The beads were briefly washed with lysis buffer with NaCl added to 300 mM. MBP-Kap114 was then eluted with buffer containing 50 mM HEPES 7.0, 50 mM NaCl, 10% (vol/vol) glycerol, 20 mM maltose, and 2 mM DTT and further purified by ion exchange using HiTrap Q HP column (Cytiva) in 25 mM Bis-Tris, pH 6.5, 0–1 M NaCl, 10% (vol/vol) glycerol, 2 mM DTT. MBP-Kap114 was subjected to a last purification step over SEC using a HiLoad Superdex 200 column (Cytiva) in Assay Buffer containing 20 mM HEPES, pH 7.4, 150 mM NaCl, 2 mM MgCl_2_, 10% (vol/vol) glycerol, and 2 mM DTT. GST-Kap114 proteins were expressed and purified as previously described (Jiou et al., 2023). In brief, the protocol is similar to the MBP-Kap114 purification described above, with lysis buffer containing 20 mM Tris-HCl, pH 7.5, 1 M NaCl, 15% (vol/vol) glycerol, 2 mM DTT, but purified with Glutathione Sepharose 4B beads (Cytiva). TEV cleavage was performed on a column and proteins were purified by SEC.

Nap1 FL (C200A, C249A, C272A mutation for specific labeling on 414C) cloned into a pHAT4 vector was a gift from Sheena D’Arcy. Nap1 mutants were generated by site-directed mutagenesis using Phusion polymerase (Thermo Fisher Scientific) or CloneAmp HiFi PCR premix (Takara Bio) with primers listed in Table S2. Nap1 FL (WT and mutants) were expressed in BL21 gold cells grown in 2X YT media, and protein expression was induced by 0.5 mM IPTG for 17 h at 18°C. Cells were harvested by centrifugation and resuspended in Nap1 lysis buffer containing 20 mM Tris, pH 7.5, 1 M NaCl, 15% (vol/vol) glycerol, 1 mM DTT, 1 mM benzamidine, 10 μg/ml leupeptin, and 50 μg/ml AEBSF. Thawed cells were lysed, and the clarified lysate was supplemented with 5 mM imidazole at pH 7.8 and added to Ni-NTA agarose (Qiagen), which was washed with the Nap1 lysis buffer with 5 mM imidazole, pH 7.8. The beads were further washed with a buffer containing 20 mM HEPES, pH 7.4, 300 mM NaCl, 15% (vol/vol) glycerol, 1 mM 2-mercaptoethanol with 25 mM imidazole, pH 7.8. Bound protein was eluted with the same buffer supplemented to 250 mM imidazole and concentrated to ∼10 ml. 1 mg of TEV protease (purified in-house) was then added to the concentrated protein, overnight, at 4°C. The protein mixture was diluted and passed through Ni-NTA beads to remove TEV and the His tag. Nap1 is further purified using HiTrap Q and Superdex S200.

Nap1 core (residues 75–365), cloned into a pET15b vector, was a gift from Karolin Luger. His-Nap1 core proteins were expressed and purified as previously described (Sarkar et al., 2020). In brief, His-Nap1 core was purified by nickel-NTA affinity and ion exchange chromatography, followed by SEC. For pull-down assays, thrombin (Cat #T4648; Sigma-Aldrich) cleavage was performed overnight at 4°C to remove the His-tag and untagged protein purified by SEC (Superdex S200 increase column [Cytiva]) in Assay Buffer.

Lyophilized *Sc* and *Xl* histones were obtained from “The Histone Source” and refolded according to established protocol (Luger et al., 1997). In brief, 4 mg of each histone was resuspended in 4 ml of unfolding buffer (7 M guanidinium HCL, 20 mM Tris-HCl, pH 7.5, and 10 mM DTT), and incubated at room temperature for 1 h. H2A and H2B, or H3 and H4, were then mixed with extra unfolding buffer to a total of 8–10 ml and incubated at room temp for another 30 min followed by dialysis with 4 liters of cold refolding buffer (2 M NaCl, 10 mM Tris-HCl, pH 7.5, 1 mM EDTA, pH 8.0, and 5 mM β-mercaptoetanol) at 4°C overnight. Refolded histones were then concentrated and purified by SEC using a HiLoad 16/600 Superdex 200 column (Cytiva) pre-equilibrated with refolding buffer. All assays in this paper were performed with *Sc* H2A-H2B unless otherwise stated (Fig. S1 E and Fig. S2 A).

*Sc* Ran^GTP^ (Gsp1 residues 1–179, with Q71L mutation to stabilize the GTP bound state) is expressed and purified in a pET21d vector as described previously with the addition of a TEV cleavage step (Fung and Chook, 2022). Briefly, Ran was expressed in BL21 gold cells, induced with 0.5 mM IPTG for 12 h at 20°C. Cells were resuspended in buffer containing 50 mM HEPES, pH 7.4, 2 mM MgOAc, 200 mM NaCl, 10 % (vol/vol) glycerol, 5 mM imidazole, pH 7.8, 2 mM 2-mercaptoethanol, 1 mM benzamidine, 10 μg/ml leupeptin, and 50 μg/ml AEBSF. Thawed cells were lysed, and the clarified lysate was incubated with Ni-NTA beads, which were washed. Ran was eluted with buffer containing 50 mM HEPES, pH 7.4, 2 mM MgOAc, 50 mM NaCl, 10% (vol/vol) glycerol, 250 mM imidazole, pH 7.8, and 2 mM 2-mercaptoethanol, concentrated, and treated with 1 mg of TEV overnight incubation at 4°C. TEV-cleaved Ran^GTP^ was purified by ion exchange using HiTrap SP HP column (Cytiva) in buffer containing 20 mM HEPES, pH 7.4, 0–1 M NaCl, 4 mM MgOAc, 10% (vol/vol) glycerol, and clean protein was flash-frozen and stored at −80°C. Ran^GTP^ activity was verified through binding assays with various importins that GTP is bound in the purified Ran, and no additional GTP loading steps were needed.

### Pull-down binding assays

All biochemical and biophysical studies were conducted at an ionic strength close to physiological salt levels of 150 mM NaCl. In vitro pull-down binding assays were performed in triplicate by incubating proteins at their indicated concentrations with 20 µl amylose resin (bead bed volume; New England Biolabs) in a 200 µl reaction in Assay Buffer (20 mM HEPES, pH 7.4, 150 mM NaCl, 2 mM MgCl_2_, 10% (vol/vol) glycerol and 2 mM DTT) for 30 min at 4°C, followed by washing with 500 µl buffer twice. Bound proteins were separated and visualized by SDS-PAGE and Coomassie Blue staining. All gels were imaged in ChemiDoc MP imaging system (Bio-rad). Nap1 band intensities were quantified using Bio-rad Image Lab software, adjusted for the intensity of the MBP-Kap114 band, and normalized to Nap1 WT control. Unpaired, two-sided Student’s *t* test was performed. Data distribution was assumed to be normal, but it was not formally tested.

### Analytical ultracentrifugation

Individual proteins were dialyzed overnight into AUC buffer containing 20 mM Tris-HCl, pH 7.5, 150 mM NaCl, 2 mM MgCl_2_ and 2 mM TCEP, at 4°C and assembled in the AUC sample chamber at the indicated concentrations in 400 µl. Sedimentation coefficients were measured by monitoring absorbance at 280 nm in a Beckman-Coulter Optima XL-1 Analytical Ultracentrifuge. Time stamps were corrected using REDATE (Zhao et al., 2013). SEDNTERP was used to calculate the buffer density, buffer viscosity, and protein partial-specific volumes (Laue et al., 1992). SEDFIT was used to calculate sedimentation coefficient distributions c(*s*) where the regularization calculated a confidence level of 0.68 was used, time-independent noise elements were accounted for, and at a resolution of 100 (Schuck, 2000). SEDFIT was also used to estimate molecular weight by obtaining the sedimentation coefficient through integration of c(*s*) and the frictional ratios by refining the fit of the model. The c(*s*) distribution and isotherm integration was done with GUSSI (Brautigam, 2015). Further isotherm fitting was performed using SEDPHAT (Zhao et al., 2015).

### Size exclusion chromatography multi-angle light scattering

SEC-MALS experiments were performed in Assay buffer without glycerol with proteins at the indicated molar ratios to 80 µM Kap114 following established protocol, including sample dialysis and buffer filtration (Sarkar et al., 2020). The concentration of proteins is high in the small injection of 100 µl to ensure complete complex formation when it is diluted in the SEC column. The data was collected and processed using ASTRA software using default settings with no de-spiking (Wyatt Technology). The run for 80 µM Kap114 + 80 µM H2A-H2B + 80 µM Nap1_2_ was interrupted at the void volume due to fractionation issues causing a total pause of 1.895 min. A correction of 1.38933 ml was applied to match other runs.

### Cryo-EM sample preparation and data collection and analysis

It is thought that Nap1_2_•H2A-H2B complex likely forms in the cytoplasm before encountering Kap114 (Bernardes and Chook, 2020; Campos et al., 2010). Therefore, we prepared a cryo-EM sample by first assembling Nap1_2_ core•H2A-H2B in the presence of excess histones to ensure binding, and then adding Kap114, before subjecting the sample to mild crosslinking. *Sc* H2A-H2B, Nap1 core, and Kap114 were dialyzed separately overnight into cryo-EM buffer containing 20 mM Tris, pH 7.5, 150 mM NaCl, and 2 mM DTT. A 1:2 M ratio mixture of Nap1 core dimer:H2A-H2B was incubated at room temperature for 10 min followed by the addition of 1 M ratio of Kap114, followed by rapid addition of glutaraldehyde to a final concentration of 0.05%. Crosslinking proceeded for 1 min before quenching and removal of glutaraldehyde by SEC using a Superdex 200 10/300 Increase column that was equilibrated with cryo-EM buffer containing TCEP (instead of DTT). 0.5 ml fractions were collected for mass photometry (MP) analysis (details below).

To assemble the Ran^GTP^/Kap114/Nap1/H2A-H2B complex, a 10 mg/ml mix of a stoichiometric molar ratio of 1 Kap114 to 1 H2A-H2B to 2 Nap1_2_ FL (His tag intact) to 1 Ran^GTP^ was dialyzed overnight before crosslinking with 0.05% glutaraldehyde for 1 min and separation by SEC in Assay buffer without glycerol. Fractions of 0.25 ml were collected for MP analysis. Fractions with the least aggregated species and most enriched with the relevant complexes (Figs. S2 and S3) were selected for grid preparation.

For Kap114/H2A-H2B/Nap1, the sample was diluted to ∼1.2 mg/ml and supplemented with 0.003125% (wt/vol) Triton X-100. For Ran^GTP^/Kap114/Nap1/H2A-H2B, the sample was diluted to a ∼1.5 mg/ml with 0.003125% (wt/vol) Tyloxapol. Quantifoil grids were glow-discharged, blotted, and plunge-frozen. Grids were screened and data was collected and analyzed as described below.

### Mass photometry for cryo-EM samples

All protein fractions were analyzed using a Refeyn TwoMP Mass Photometer. The laser was warmed up for an hour before use. During that waiting period, the glass slide was washed by alternating between Milli-Q filtered water, isopropanol, and then Milli-Q water, twice. A 2 by 4 strip of wells was adhered to the middle of the glass slide. Immersion oil was applied to the lens before placing the glass slide on top. Prior to the measurement, 2 mg/ml BSA was diluted 100-fold with freshly filtered buffer from the buffer used in S200 of the cryo-EM complex. BSA was used for generating the calibration curve, using an additional 10-fold dilution: we first added 16.2 µl of buffer into a well and brought it to focus then mixed in 1.8 µl of BSA and 60 s movies were recorded. Fractions were diluted to under 7,000 counts for collection in a similar manner as the BSA. Data were processed through Gaussian fitting to provide the peak mass and relative populations using the Refeyn analysis software.

### Cryo-EM data collection

A 48-h data collection with the best grid of the Kap114/H2A-H2B/Nap1 core complex was performed at the UT Southwestern Cryo-Electron Microscopy Facility (CEMF) on a Titan Krios microscope (Thermo Fisher Scientific) at 300 kV with a Gatan K3 detector in correlated double sampling super-resolution mode at a magnification of 105,000X corresponding to a pixel size of 0.415 Å. A total of 10,806 movies were collected; each movie was recorded for a total of 60 frames over 5.4 s with an exposure rate of 7.8 electrons/pixel/s. The datasets were collected using SerialEM software (Mastronarde, 2005) with a defocus range of −0.9 and −2.4 µm.

A 24-h data collection was performed on the best grid with Kap114/H2A-H2B/Nap1 FL/Ran^GTP^ complex at the UTSW CEMF on a Titan Krios 300 kV microscope equipped with a Falcon 4i detector at a magnification of 165,000X at pixel size of 0.738 Å with defocus range of −0.9 to −2.2 µm. A total of 9,331 movies were recorded for a total of 1,114 frames over 3.6 s with an exposure rate of ∼8 electrons/pixel/s.

### Cryo-EM data processing

Cryo-EM data for the Kap114/H2A-H2B/Nap1 core complex was processed using cryoSPARC version 3 (Punjani et al., 2017). The movies were subjected to Patch Motion Correction (binned twice) and Patch CTF Estimation. Training particles were obtained by blob picking on 100 micrographs and followed by 2D classification of the particle stack. Particles containing Kap114 were used to train on Topaz (Bepler et al., 2019), which picked 665,443 particles. These particles were subjected to three rounds of 2D classification (300 classes/run). 113,471 particles that contained both Kap114 and Nap1 were split into two Ab-initio classes, the Nap1_2_•H2A-H2B•Kap114 and Nap1_2_•Kap114•H2A-H2B, and further classified using Heterogenous Refinement. 54,170 particles of Nap1_2_•H2A-H2B•Kap114 and 59,310 particles of Nap1_2_•Kap114•H2A-H2B were further refined using Non-Uniform (NU) Refinement resulting in 4.02 and 3.65 Å maps, respectively. To obtain more particles, these maps were used to generate templates for additional template picking on cryoSPARC. This resulted in 4,314,112 total particles that contained Nap1_2_ only, Kap114•H2A-H2B, Nap1_2_•H2A-H2B•Kap114, and Nap1_2_•Kap114•H2A-H2B. After the first round of 2D classification ∼1 million particles were further processed for Nap1 core dimer, and another ∼1 million particles were further processed for the Kap114-containing complexes. Both Nap1 and Kap114 complexes underwent three more rounds of additional 2D classifications to obtain 468,627 and 675,417 particles, respectively. Two ab initio classes were generated for Nap1 particles and four ab initio classes were generated for the Kap114 complexes followed by classification with heterogeneous refinement. 230,210 Nap1 particles were used in NU Refinement resulting in the final map with 3.2 Å resolution. The Nap1_2_•H2A-H2B•Kap114 and Nap1_2_•Kap114•H2A-H2B particles obtained from Topaz were merged with ones obtained from template picking. Duplicates were then removed, resulting in 136,011 Nap1_2_•H2A-H2B•Kap114 and 148,410 Nap1_2_•Kap114•H2A-H2B final particles that were used for reconstruction in Non-uniform (NU) refinement with default parameters to obtain maps with 3.5 and 3.2 Å, respectively. Local refinement was performed using a custom fulcrum position at the center of h19 helices, determined in ChimeraX, to obtain improved maps for Nap1 in both reconstructions. Additionally, for Nap1_2_•Kap114•H2A-H2B map, pose/shift Gaussian prior to alignment was used.

Cryo-EM data for the Kap114/H2A-H2B/Nap1 FL/Ran^GTP^ complex was processed using cryoSPARC version 4.3. The movies were subjected to Patch Motion Correction (no binning) and Patch CTF Estimation. Blob picking on all of the micrographs yielded 2,567,783 initial particles, which was followed by five rounds of 2D classification to 306,757 particles, which were submitted to ab initio reconstruction and hetero-refinement to obtain four classes. Two classes containing Kap114 bound to Ran^GTP^ were used as training particles for Topaz to pick 1,381,753 particles, which were cleaned by three rounds of 2D classification to 358,680 particles. These particles were submitted to ab initio reconstruction and hetero-refinement to obtain four classes, one of which was used for further local CTF refinement and NU refinement with default parameters to obtain a map with 2.9 Å refinement. Density for Nap1_2_ and H2A-H2B was present in hetero-refinement map and early iterations for NU refinement, but not in the final map. Therefore, a mask was generated using a Nap1_2_•H2A-H2B•Kap114 docked onto the low-resolution map, and local refinement was performed with default parameters. Directional FSCs were generated using 3DFSC server (Tan et al., 2017).

### Structure building/modeling

The initial Nap1 model generated with AlphaFold-Multimer was used to build into the cryo-EM structure of the Nap1_2_ (Evans et al., 2022, *Preprint*). The initial models used to build the Nap1_2_•Kap114•H2A-H2B structure included Kap114•H2A-H2B (PDB:8F0X), the Alpha-Fold model of Kap114 (AF-P53067-F1), and our cryo-EM structure of the Nap1 dimer. The Nap1_2_•H2A-H2B•Kap114 was built using the Ran^GTP^•H2A-H2B•Kap114 cryo-EM structure (PDB: 8F1E), AF-P53067-F1, and our cryo-EM model of the Nap1 dimer as initial models. The Nap1_2_•H2A-H2B•Kap114•Ran^GTP^ structure was built using the Nap1_2_•H2A-H2B•Kap114 cryo-EM structure determined in this study and the Ran^GTP^•H2A-H2B•Kap114 (PDB: 8F1E) (Jiou et al., 2023). All initial models were roughly docked into the map using UCSF Chimera or ChimeraX (Pettersen et al., 2004, 2021) and then subjected to real-space refinement with global minimization and rigid body restraints in Phenix (Adams et al., 2010). The resulting structures were then manually rebuilt and refined using Coot (Emsley, 2018), further corrected using ISOLDE (Croll, 2018) on UCSF ChimeraX, and subjected to the last round of refinement in Phenix. We used PDBe PISA to calculate solvent-accessible surface areas (Krissinel and Henrick, 2007). We also used PyMOL version 2.5 and the APBS electrostatic plugin for 3D structure and electrostatic analysis (Jurrus et al., 2018; PyMOL, 2020).

### Fluorescence polarization

To generate fluorescently labeled Nap1 FL, the proteins were treated with 1 mM DTT for 30 min at room temperature and buffer-exchanged into 20 mM HEPES, pH 7.4, 500 mM NaCl, 10% (vol/vol) glycerol using a HiTrap Desalting column (Cytiva). 4 M excess XFD488 (ATT Bioquest) was added and reaction incubated for 2 h at room temperature in the dark before removal of excess dye by SEC (Superdex S200 increase) in assay buffer. Fluorescence polarization assays were performed in Assay Buffer in triplicates of sixteen 20 μl samples. *Sc* H2A-H2B was serially diluted from 4 µM and mixed with 20 nM labeled Nap1_2_ proteins in a 1:1 ratio to yield final concentrations in a 384-well black bottom plate (Corning). Measurements were performed in a CLARIOstar Plus plate reader (BMG Labtech) with top optics using excitation filter 482-16, dichroic filter LP 504, and emission filter 530-40; 50 flashes per well. The gain was optimized to target an mP of ∼200. Data was analyzed in PALMIST (Scheuermann et al., 2016) and plotted in GUSSI.

### DNA competition assays

601 Widom DNA was purified from pUC19-30X601 grown in C2925 dam^-^dcm^-^ cells (gift from Dr. Mike Rosen) using Qiagen Giga prep kit following the manufacturer’s protocol. The DNA was resuspended in 10 mM Tris-HCl, pH 7.4, 1 mM EDTA in the last step of purification. EcoRV (New England Biolabs) cleavage was performed in CutSmart buffer overnight and the DNA was extracted using PCIAA and precipitated in sodium acetate, pH 5.2, and ethanol. The DNA was then rinsed with ethanol, dried, and resolubilized in 10 mM Tris-HCl pH7.4, 10 mM MgCl_2_. The cut insert was separated from the vector using PEG8000 precipitation and subjected to a final ion exchange purification on a DEAE-FF column before it was concentrated and frozen.

DNA was mixed with H2A-H2B, Kap114, and/or Nap1_2_ FL with and without Ran^GTP^ at the indicated concentrations in 15 µl reactions in assay buffer. DNA was either premixed with H2A-H2B in a 3X concentration master mix or, alternatively, added at the end. Reactions were incubated at room temperature for 30 min and then 5 µl of sample buffer containing 20% (vol/vol) glycerol and bromophenol blue was added. 8 µl each of this gel sample was run on two separate Native 5% PAGE gels with 0.5X TBE at 150 V for 40 min. One gel was stained with EtBr in water and the other with Coomassie Blue. Gels were imaged in ChemiDoc MP imaging system. Assays were performed in duplicates.

### Nucleosome assembly assays

Tetrasomes were made by mixing *Xl* H3-H4 tetramers and DNA at 1:1 ratio (DNA conc = 1 mg/ml) in 10 mM HEPES pH7.4, 1 mM EDTA, 2 M NaCl, 1 mM DTT and then dialyzed step-wise from 2 M NaCl (0.5 h) to 1 M (1 h), 0.85 M (1.5 h), 0.65 M (1.5 h), 0.2 M (1.5 h), and 0.15 NaCl M (overnight) at 4°C. The volume was measured at the end and the reaction was diluted with assay buffer before use, assuming 100% formation of tetrasomes. Master mixes of *Sc* H2A-H2B, Kap114, and Nap1_2_ FL with or without Ran^GTP^ were assembled and mixed with tetrasomes in 10 µl reactions on ice and incubated at room temperature for 1 h before addition of 4X sample buffer with 20% glycerol. 3 µl of the resulting samples were visualized the same way as DNA competition assays. Assays were performed in duplicates. The input sample in lane 1 of Fig. 6 A shows that tetrasome formation is not 100% complete and free DNA was present. The strongest nucleosome band is observed in lane 7 in Fig. 6 A with 6 µM Nap1_2_•H2A-H2B, suggesting that the tetrasome concentration was likely ∼3 µM.

### Online supplemental material

Fig. S1 shows the biochemical analysis of Kap114, Nap1, and H2A-H2B binary and tertiary interactions. Fig. S2 shows the biochemical analysis of Kap114, Nap1, and H2A-H2B in the presence of Ran^GTP^ and details for cryoEM sample preparation and data analysis for the quaternary complex. Fig. S3 shows cryoEM sample preparation and data analysis for the ternary Kap114/Nap1/H2A-H2B sample. Fig. S4 shows the structural analysis for complexes described in this study and control binding experiment for Nap1 mutants. Table S1 shows cryoEM data collection, refinement, and validation statistics for the Nap1 core and Nap1_2_•Kap114•H2A-H2B structure. Table S2 contains primers used in this study.

## Supplementary Material

Table S1 shows cryoEM data collection, refinement and validation statistics for the Nap1 core and Nap1_2_•Kap114•H2A-H2B structure.

Table S2 contains primers used in this study.

SourceData F1 is the source file for Fig. 1.

SourceData F3 is the source file for Fig. 3.

SourceData F4 is the source file for Fig. 4.

SourceData F5 is the source file for Fig. 5.

SourceData F6 is the source file for Fig. 6.

SourceData FS1 is the source file for Fig. S1.

SourceData FS2 is the source file for Fig. S2.

## Data Availability

The data underlying Figs. 2, S2, and S3 are openly available in PDB/EMDB at 9B3I/EMD-44141, EMD44150, and EMD44151 (Fung et al., 2024a, 2024b, 2024c); 9B31/EMD-44120, EMD-44121, and EMD-44122 (Jiou et al., 2024c); 9B3F/EMD-44136, EMD-44137, and EMD-44140 (Jiou et al., 2024b); and 9B23/EMD-44095 (Jiou et al., 2024a).
