# Supplementary material for: Nap1 and Kap114 co-chaperone H2A-H2B and facilitate targeted histone release in the nucleus
Source: J Cell Biol. 2024 Nov 27;224(1):e202408193. doi: 10.1083/jcb.202408193 (PMC11602657; doi:10.1083/jcb.202408193)
Supplement: Table S1 — shows cryoEM data collection, refinement and validation statistics for the Nap1 core and Nap12•Kap114•H2A-H2B structure. [file JCB_202408193_TableS1.docx]

**Table S1. Cryo-EM data collection, refinement, and validation statistics continued.**

|  | Nap1_2_ | Nap1_2_•*Kap114*•H2A-H2B | | |
| --- | --- | --- | --- | --- |
|  | PDB: 9B23  EMD-44095 | Consensus Map  EMD-44122 | Locally refined map for Nap1_2_  EMD-44121 | Composite Map  PDB: 9B31  EMD-44120 |
| Data collection and processing | | | | |
| Magnification (X) | 105,000 | | | |
| Voltage (kV) | 300 | | | |
| Electron exposure (e^–^/Å^2^) | 52 | | | |
| Defocus range (μm) | 1.5-2.5 | | | |
| Pixel size | 0.83 | | | |
| Symmetry (Å) | C1 | | | |
| Initial particle images | 4,314,112 | | | |
| Final particle no. | 230,210 | 148,410 | | |
| Map resolution (Å) | 3.21 | 3.20 | 4.84 |  |
| FSC threshold | 0.143 | | | |
| Refinement | | | | |
| Initial model used  (PDB code) | AlphaFold-multimer Nap1 FL model |  |  | AF-P53067-F1, 8F0X, 9B23 |
| Model composition |  |  |  |  |
| Non-H Atoms | 4,670 |  |  | 13,559 |
| Protein residues | 568 |  |  | 1,682 |
| Mean *B* factors (Å^2^) | 196.99 |  |  | 129.6 |
| R.m.s. deviations |  |  |  |  |
| Bond lengths (Å) | 0.003 |  |  | 0.004 |
| Bond angles (°) | 0.592 |  |  | 0.543 |
| CCvolume/mask | 0.68/0.69 |  |  | 0.76/0.75 |
| Validation | | | | |
| MolProbity score | 1.69 |  |  | 1.30 |
| Clashscore | 10.54 |  |  | 5.54 |
| Poor rotamers (%) | 0 |  |  | 0 |
| Ramachandran plot |  |  |  |  |
| Favored (%) | 97.16 |  |  | 98.26 |
| Allowed (%) | 2.84 |  |  | 1.74 |
| Disallowed (%) | 0 |  |  | 0 |
| CaBLAM outliers (%) | 1.25 |  |  | 1.15 |
| EMRinger score | 0.94 |  |  | 1.87 |
