# Supplementary material for: Nap1 and Kap114 co-chaperone H2A-H2B and facilitate targeted histone release in the nucleus
Source: J Cell Biol. 2024 Nov 27;224(1):e202408193. doi: 10.1083/jcb.202408193 (PMC11602657; doi:10.1083/jcb.202408193)
Supplement: Table S2 — contains primers used in this study. [file JCB_202408193_TableS2.docx]

| Purpose | Primer |
| --- | --- |
| Kap114_Y939A/D942A_F | GTTGAAAGAAGCTATTGATGCTGATGTCGATGA |
| Kap114_Y939A/D942A_R | TCATCGACATCAGCATCAATAGCTTCTTTCAAC |
| Kap114_D928A/D929A_F | GGGAAGATGTAGCTGCTGTACTCGATTAC |
| Kap114_ D928A/D929A_R | GTAATCGAGTACAGCAGCTACATCTTCCC |
| Kap114_Δ19L_F | GGTGGCTCTGGCGGTTCCTCTGTTGTTCAGCTAC |
| Kap114_Δ19L_R | GGAACCGCCAGAGCCACCACTTTGGAAGCTTAACT |
| Nap1_E194A_F | AACCGCGTTAGCGAACTTGCCC |
| Nap1_D201A/D205A_R | GGCAGTGATCGTGGCGGCAACG |
| Nap1_D205A_F | CACGATCACTGCCCGCGATGCG |
| Nap1_D205A_R | CGCATCGCGGGCAGTGATCGTG |
| Nap1_Q292A_F | [Phos]CAAGGCGAGAAACAAGACCAC |
| Nap1_E288A/R290A_R | [Phos]GCCATTGCTAGGTCCACAGT |

**Table S2. Oligos and sequencing primers used in this study.**
