# Supplementary figures and images for: Nap1 and Kap114 co-chaperone H2A-H2B and facilitate targeted histone release in the nucleus
Source: J Cell Biol. 2024 Nov 27;224(1):e202408193. doi: 10.1083/jcb.202408193 (PMC11602657; doi:10.1083/jcb.202408193)

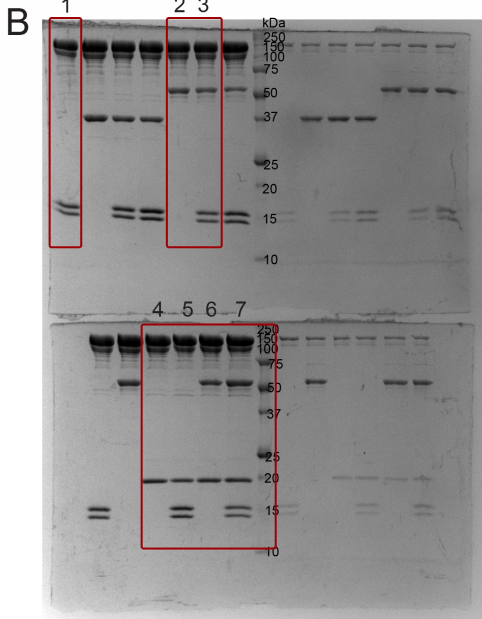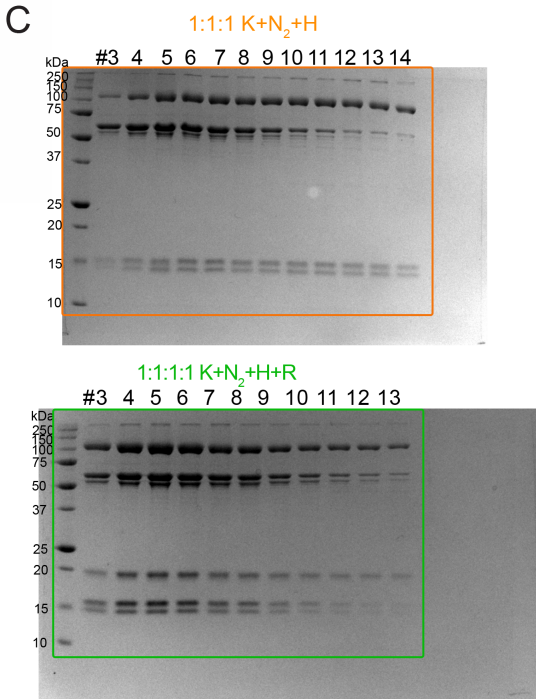

Supplement: SourceData F1 — is the source file for Fig. 1. [file JCB_202408193_SourceDataF1.pdf]

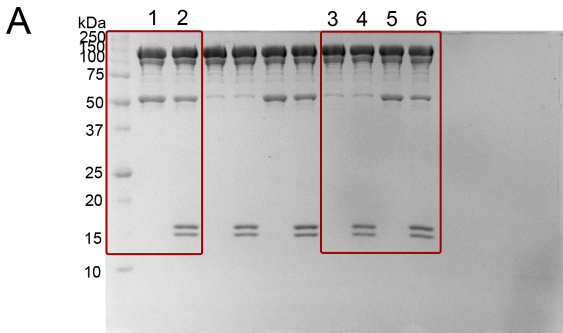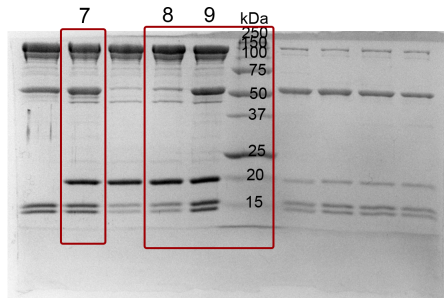

**B, left**

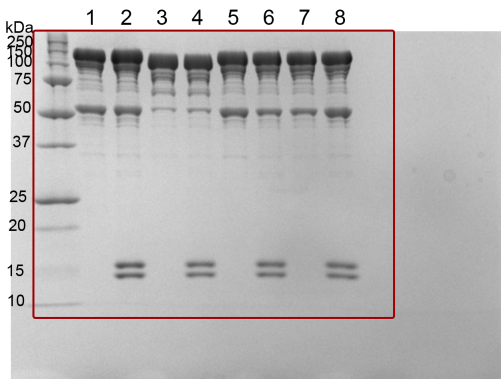

**B, right**

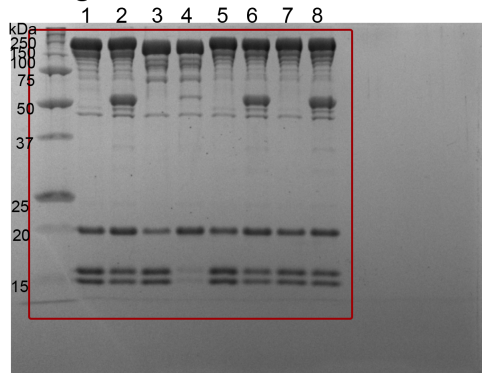

Supplement: SourceData F3 — is the source file for Fig. 3. [file JCB_202408193_SourceDataF3.pdf]

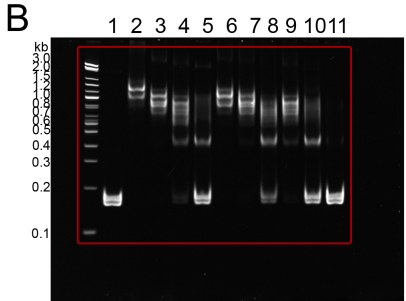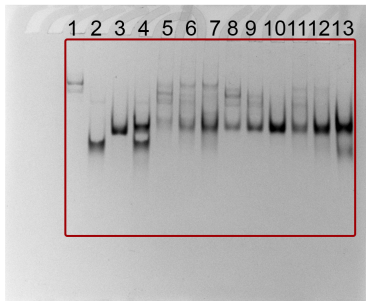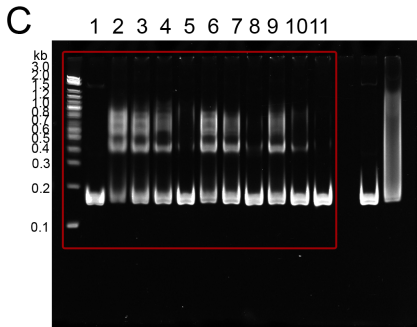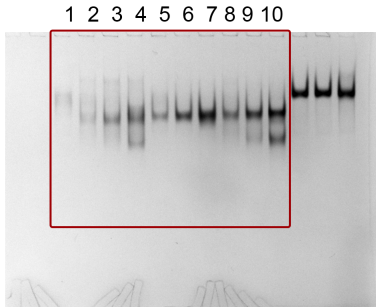

Supplement: SourceData F4 — is the source file for Fig. 4. [file JCB_202408193_SourceDataF4.pdf]

**B**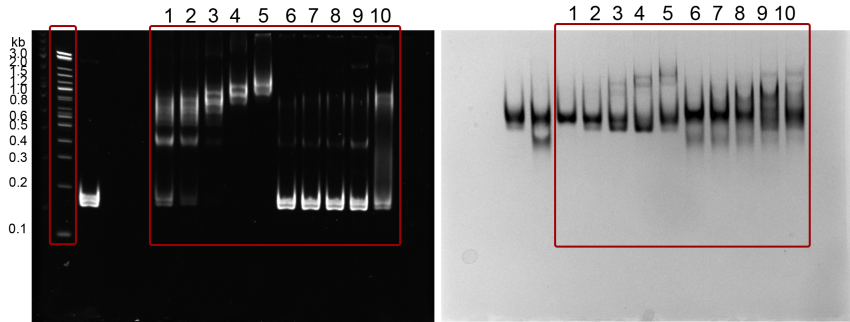**C**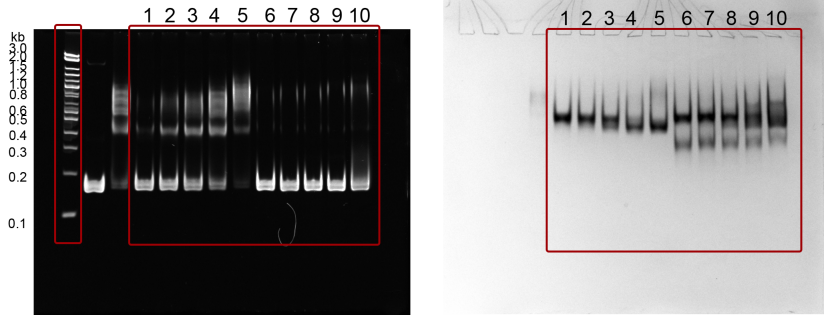

Supplement: SourceData F5 — is the source file for Fig. 5. [file JCB_202408193_SourceDataF5.pdf]

A

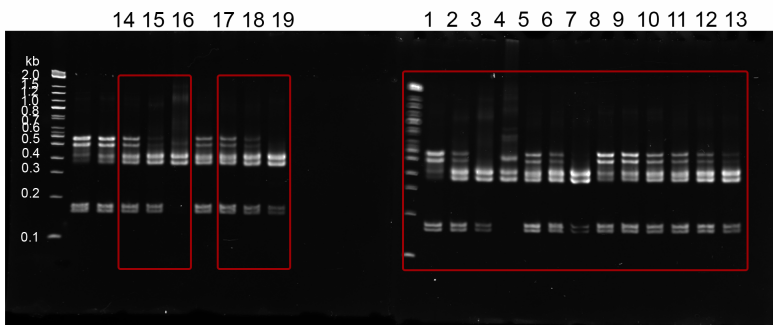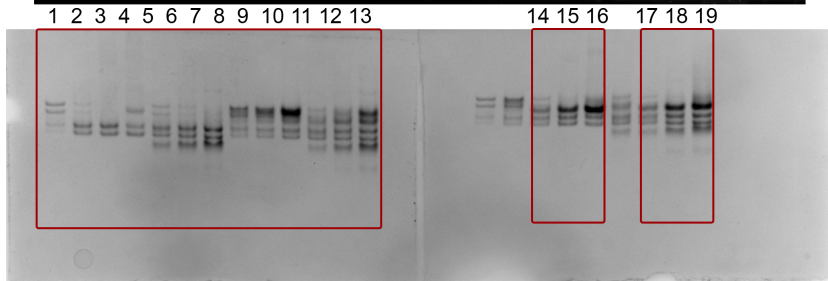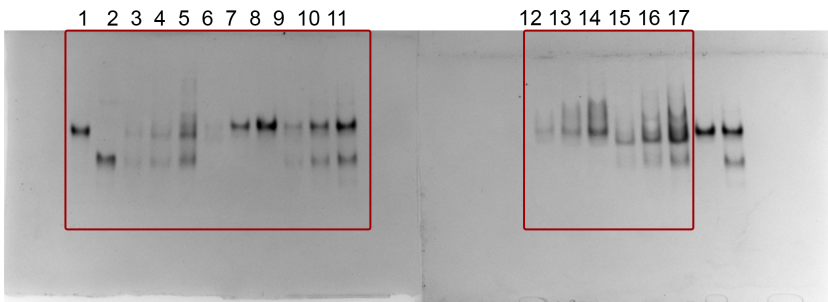

Supplement: SourceData F6 — is the source file for Fig. 6. [file JCB_202408193_SourceDataF6.pdf]

**E**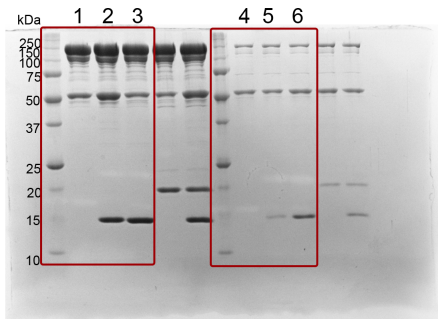**F**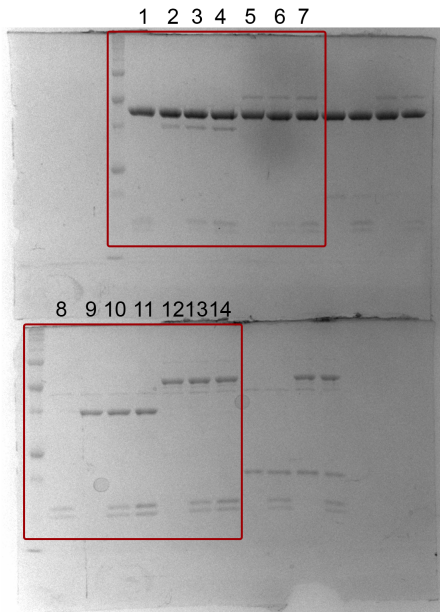

Supplement: SourceData FS1 — is the source file for Fig. S1. [file JCB_202408193_SourceDataFS1.pdf]

A, left

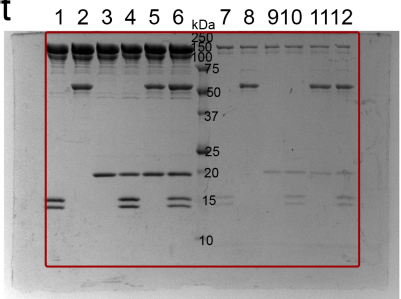

A, right

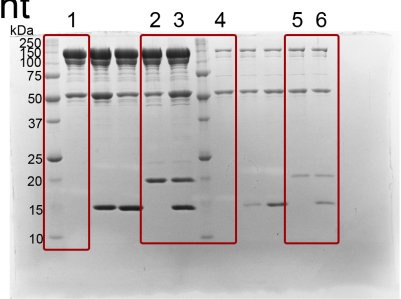

B

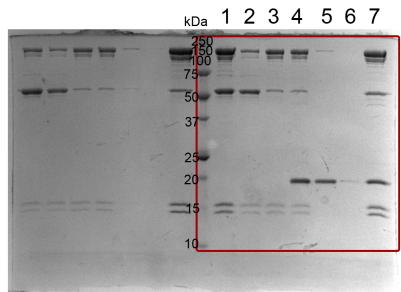

Supplement: SourceData FS2 — is the source file for Fig. S2. [file JCB_202408193_SourceDataFS2.pdf]
